# Reliably assessing the electronic structure of cytochrome P450 on today’s classical computers and tomorrow’s quantum computers

**DOI:** 10.1073/pnas.2203533119

**Published:** 2022-09-12

**Authors:** Joshua J. Goings, Alec White, Joonho Lee, Christofer S. Tautermann, Matthias Degroote, Craig Gidney, Toru Shiozaki, Ryan Babbush, Nicholas C. Rubin

**Affiliations:** ^a^Google Quantum AI, Google Research, Venice, CA 90291;; ^b^Quantum Simulation Technologies, Inc., Boston, MA 02135;; ^c^Department of Chemistry, Columbia University, New York, NY 10027;; ^d^Medicinal Chemistry, Boehringer Ingelheim Pharma GmbH & Co KG, 88397 Biberach, Germany;; ^e^Department of General, Inorganic, and Theoretical Chemistry, University of Innsbruck, 6020 Innsbruck, Austria;; ^f^Quantum Lab, Boehringer Ingelheim, 55218 Ingelheim am Rhein, Germany

**Keywords:** quantum computing, quantum chemistry, fault-tolerant quantum algorithms

## Abstract

An accurate assessment of how quantum computers can be used for chemical simulation, especially their potential computational advantages, provides important context on how to deploy these future devices. To perform this assessment reliably, quantum resource estimates must be coupled with classical computations attempting to answer relevant chemical questions and to define the classical algorithms simulation frontier. Herein, we explore the quantum computation and classical computation resources required to assess the electronic structure of cytochrome P450 enzymes (CYPs) and thus define a classical–quantum advantage boundary. This is accomplished by analyzing the convergence of density matrix renormalization group plus *n*-electron valence state perturbation theory (DMRG+NEVPT2) and coupled-cluster singles doubles with noniterative triples [CCSD(T)] calculations for spin gaps in models of the CYP catalytic cycle that indicate multireference character. The quantum resources required to perform phase estimation using qubitized quantum walks are calculated for the same systems. Compilation into the surface code provides runtime estimates to compare directly to DMRG runtimes and to evaluate potential quantum advantage. Both classical and quantum resource estimates suggest that simulation of CYP models at scales large enough to balance dynamic and multiconfigurational electron correlation has the potential to be a quantum advantage problem and emphasizes the important interplay between classical computations and quantum algorithms development for chemical simulation.

## Significance

Chemical simulation is one of the most promising applications for future quantum computers. It is thought that quantum computers may enable accurate simulation for complex molecules that are otherwise impossible to simulate classically; that is, it displays quantum advantage. To better understand quantum advantage in chemical simulation, we explore what quantum and classical resources are required to simulate a series of pharmaceutically relevant molecules. Using classical methods, we show that reliable classical simulation of these molecules requires significant resources and therefore is a promising candidate for quantum simulation. We estimate the quantum resources, both in overall simulation time and the size. The insights from this study pave the way for future quantum simulation of complex molecules.

Chemical simulation is among the most promising applications of quantum computers. Despite this, it remains a challenge to accurately assess and identify chemical problems for which one can reasonably expect future quantum computational advantage. This problem is challenging for many reasons, but two key difficulties emerge when demarcating the boundary of quantum computational advantage. First, given the myriad conventional polynomial-scaling electronic structure methods, it is difficult to find chemical problems that will not yield to at least one classical method. For any claim that a problem is classically difficult—or even impossible—there is no guarantee that the claim will not be challenged by a new method at some time later. The second difficulty is that quantum algorithms for chemistry are still an active area of development, so estimates of the resources required to compile and run experiments on quantum computers will continue to evolve. Until the development of a truly scalable and fault-tolerant quantum computer, resource estimates of the most promising quantum algorithms are limited to rigorous calculations of prefactors yielding runtime upper bounds. Moreover, a chemical problem may prove to be prohibitive on either a quantum or a classical computer.

Thus, any attempts to determine the boundary of quantum computational advantage must involve high-accuracy classical quantum chemistry simulations along with a detailed resource estimation of quantum algorithms and the cost of measuring chemically relevant observables. Ideally, quantum advantage is defined within a realistic model of chemistry and is associated with a computation that answers a typical chemistry question. In this work, we articulate the nuances in describing this boundary concretely by focusing on the quantum and classical resources required to reliably simulate the active space of a biologically important enzyme. We simulate the active space of cytochrome P450 (CYP) mimics with a variety of classical electronic structure methods to assess the degree of strong correlation and what would be required to evaluate 1) the chemical mechanism of reactivity for CYPs and 2) the spin-state ordering of reactive intermediates of the catalytic cycle of CYPs that are necessary for a correct description of energy barriers. To assess the quantum cost we evaluate runtimes and logical qubit requirements required to implement phase estimation within the surface code error correction scheme.

We focus on costing out these two questions as they are representative of the types of chemical questions one could ask about a realistic system that highlight the difficulties of application of quantum and classical algorithms in chemical science. To this end, we use several systematically improvable classical electronic structure methods (discussed below) to scope out the limits of classical algorithms to obtain reliable chemical energetics. Having shown that classical calculations will be cost prohibitive, we compute the resource estimates for quantum phase estimation algorithms, which would be required for efficient and reliable computation of the energetics in CYP mimics. What emerges from these detailed accountings of cost is that despite a potential exponential simulation time advantage for quantum computers, direct simulation of a large enough system to reliably account for dynamic correlation may be beyond the reach of classical and quantum devices. This suggests room for further development of quantum algorithms.

To quantify the cost, and therefore the limits, of classical electronic structure calculations we focus on families of methods that are systematically improvable. Such methods have the property that one can estimate convergence of any given property toward the exact result, which is the basis of truly predictive computation. One such family of methods is the coupled-cluster (CC) hierarchy (1–7) that, for most systems, provides results with controllable error at a high, but polynomial, cost. For systems with significant multireference character, coupled-cluster methods will fail, and one must turn to multireference methods usually based upon an exact or near-exact solution within an active space of orbitals. In theory, such methods can be systematically improved by increasing the size of the active space, but in practice the high cost often necessitates small active spaces or aggressive truncations to the molecular model for which the error is difficult to control. For small active spaces, the full configuration interaction (FCI) calculation can be performed directly, while for larger active spaces one must turn to approximate methods such as the density matrix renormalization group (DMRG) (8–11), full configuration interaction quantum Monte Carlo (FCIQMC) (12), or some variant of selected configuration interaction method (13–16). Although research into these methods has enabled large active-space calculations in recent years, medium- to large-sized molecular systems will still require some treatment of electron correlation outside of the active space to obtain qualitatively correct results. Progress has been made with various flavors of multireference perturbation theory (17, 18) and multireference coupled-cluster theory (19), but a balanced description of electron correlation outside of the active space remains a major challenge.

The quantum algorithm we choose to compare against is phase estimation, which allows one to sample in the eigenbasis of a given Hamiltonian. Assuming one can prepare an initial state with sufficient overlap with the ground state, phase estimation can be used to estimate the ground-state energy of a chemical system. Although there are a variety of quantum algorithms for acquiring different types of chemical observables, energy computations provide direct comparison to address the aforementioned problem of spin-gap estimation with single-point energy calculations. Improvements to the algorithm have brought costs down from prohibitively high runtimes to scalings that now scale linearly in inverse precision and the square root of the basis-independent information content of the Hamiltonian (20). However, asymptotic scalings are not enough to define a quantum advantage boundary. Prefactor estimates along with compilation considerations have been taken into account to provide an upper bound to a real-time estimate for the runtime of a quantum algorithm subject to the assumption of high initial overlap. To make a direct comparison to classical simulation costs in active spaces of chemical systems, we perform a quantum resource estimation assuming phase estimation of the qubitized quantum walk operator (20). Qubitization strongly depends on the type of tensor factorization one uses for the two-electron integrals in the standard electronic structure Hamiltonian. We study this dependency for three different factorizations and demonstrate that tensor hypercontraction leads to substantially lower runtimes in CYP systems. Finally, we analyze tradeoffs in qubit count and number of Toffoli factories to define a quantum advantage frontier for a variety of hardware configurations.

To compare cost of classical and quantum computation, we focus on models of the active site of CYP proteins. Compared to exotic systems, like the FeMo cofactor of nitrogenase (FeMoco) (20), that are usually used for quantum resource estimates, the active sites of CYP proteins are more representative of the typical systems that can benefit from chemical simulation. The superfamily of CYPs is membrane-bound heme-containing enzymes that function mostly as monooxygenases. In the human genome 57 CYP isoforms are encoded; moreover, it is the largest family of hemoproteins known, with more than 300,000 members throughout all organisms (21). The major role of CYPs lies in the detoxification of organisms. The most common detoxification mechanism involves a single-oxygen insertion into C-H bonds of CYP substrates, thereby generating a hydroxy group, which enables further metabolism. The oxidation by CYPs is also the most common metabolization mechanism for drugs in humans, where more than 70% of all drugs are metabolized by just two CYP isoforms (CYP 3A4 and CYP 2D6) (22). In the case of CYP 3A4, which is known to roughly metabolize 50% of all marketed drugs (23), more than one substrate molecule may be accommodated in the active site (24).

**Fig. 1.**
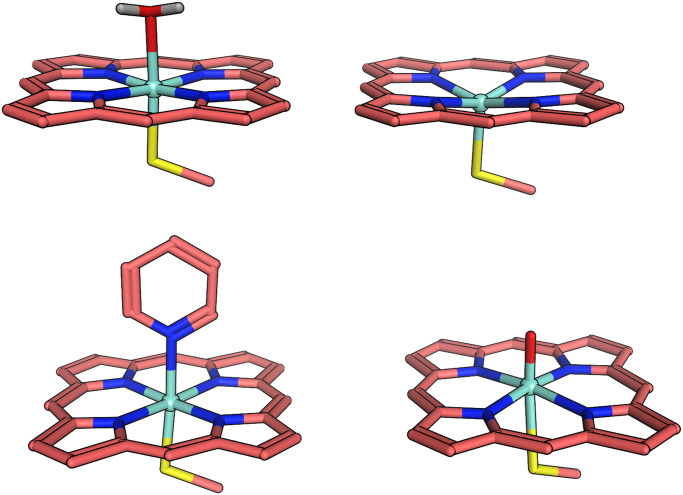
Model systems employed in this study. (Top Left) The resting state with water bound to heme. (Top Right) The pentacoordinated “empty” state. (Bottom Left) Pyridine inhibitor bound model complex. (Bottom Right) Cpd I. To improve clarity all nonpolar hydrogen atoms are hidden.

The oxidation by CYPs is a multistep catalytic cycle, shown in *SI Appendix*, section 2, Fig. 1, involving at least eight intermediates (25), consuming one molecule of O2, two electrons, and two protons to achieve the monooxygenation of a substrate. The catalytically active species, compound I (Cpd I) (25), is thought to be a polyradical neutral species involving an iron porphyrin ring coordinating to atomic oxygen and thiolate from cysteine. The spin-state energetic orderings of Cpd I are still a matter of debate, where nearly degenerate doublet and quartet states are postulated (25). The spin states are sensitive to the protein environment and Cpd I is often referred to as a “chameleon species,” as it changes its spin nature depending on subtle structural changes in the protein. A complete theoretical description of this catalytic cycle is challenging because incorrect results for a single intermediate can qualitatively change the chemistry. If a single intermediate has an electronic structure demanding a more accurate treatment, then for consistency all intermediates should be calculated at this level of theory. To portray the classical and quantum computing costs along the CYP catalytic cycle, we decided to employ model systems of the water-bound resting state, the pentacoordinate “empty” state, and Cpd I. Additionally, due to the high pharmaceutical relevance, we also include a model system corresponding to an inhibitor-bound active site of P450, where a pyridine is bound to the heme–iron, such as shown in Fig. 1. This model system has proved to be very useful in the investigation of reaction mechanisms of Cpd I by density functional theory (DFT) either in vacuum or as a quantum mechanical (QM) region in quantum mechanics/molecular mechanics (QM/MM) calculations (26, 27). Moreover, the truncation to the model system does not cause lack of generality, as Lonsdale et al. (28) demonstrated the electronic structures of Cpd I to be very similar within human CYP isoforms. However, to achieve an accurate description of individual isoforms and substrate interaction requires multiscale modeling (29–31), as CYP action has been shown to have subtle dependence on the protein environment (28, 32), especially on polarization, hydrogen bonding, and membrane composition.

In this work we analyze the model compounds with state-of-the-art classical electronic structure methods, estimate the quantum resources required for different-sized active-space Hamiltonians, and provide a classical characterization of the electron correlation in these systems. In *Section 1* we describe the series of active-space models studied and DMRG calculations with an *n*-electron valence state perturbation theory (NEVPT2) correction to determine accurate spin gaps and discuss potential hero calculations that can be performed to resolve the spin-gap problem. In *Section 2* we provide quantum resource estimates and runtimes after compiling to a surface-code quantum error correction scheme. With full compilations to realistic hardware configurations we compare runtimes from DMRG and the quantum computer for the task of simulating the ground-state energy of the active-space models. For complete analysis, in *SI Appendix*, section 6 we characterize the electronic structure of four model CYP compounds and demonstrate that the empty, inhibited, and resting states can be characterized by single-reference electronic structure methods while Cpd I exhibits some multiconfigurational character. Specifically, we demonstrate that traditional metrics for strong correlation, $\text{max}(|t_{1}|)$-diagnostic, and spin contamination, are corrected by including dynamic correlation and thus the three aforementioned compounds are classified as “artificially” symmetry broken. We close with a discussion of future research directions and chemically relevant observables for characterizing CYP with quantum or classical computation.

## 1. Classical Calculations of Electron Correlation in P450 Models

The energetics, dominant electronic structure features, and spin gaps have been the subjects of many quantum chemistry calculations on various CYP isoforms (25, 33–36), ranging from full-space DFT calculations (37–40) to active-space models with dynamic correlation corrections (41–43). The model systems in this work were derived from experimental X-ray structures of CYP3A4 by removing all noniron coordinating entities of the protein and the solvent. Further details on how the geometries were determined can be found in *SI Appendix*, section 2.1. In the following section we describe the construction of a hierarchy of active spaces and use coupled-cluster singles doubles with noniterative triples [CCSD(T)] and DMRG with NEVPT2 corrections to characterize the electronic structure at various experimentally motivated spin states. The DMRG timings and accuracy are used to provide context for the quantum resource estimates and ultimately motivate a potential quantum advantage boundary. The computational details for all calculations can be found in *SI Appendix*, section 3.

### Active-Space Selection.

To create the active-space models, the orbitals of the high-spin restricted open-shell Hartree-Fock (ROHF) state in the correlation-consistent polarized valence double zeta (cc-pVDZ) basis (44) were localized with the Pipek–Mezey localization scheme (45) to yield a set of local orbitals for each compound. We constructed active spaces of increasing size in a hierarchical manner by starting with the five singly occupied orbitals of the high-spin reference (A) and then adding orbitals from the occupied and virtual spaces as summarized in *SI Appendix*, section 8, Table 3. For heme–iron systems the computed spin gap will depend strongly on the choice of active space (42, 46–52). While most previous studies sought the most efficient possible active space of a given size, our strategy is designed to yield a balanced hierarchy of active spaces that will facilitate analysis of computational cost and convergence to the exact limit.

This process was repeated for each of the four compounds to yield a hierarchy of active spaces (A, B, C, D, E, F, G, X), each a superset of the previous, all with roughly the same filling fraction. The numbers of orbitals and electrons in each active space are tabulated in *SI Appendix*, section 8.

### Spin-State Ordering from DMRG+NEVPT2.

**Fig. 2.**
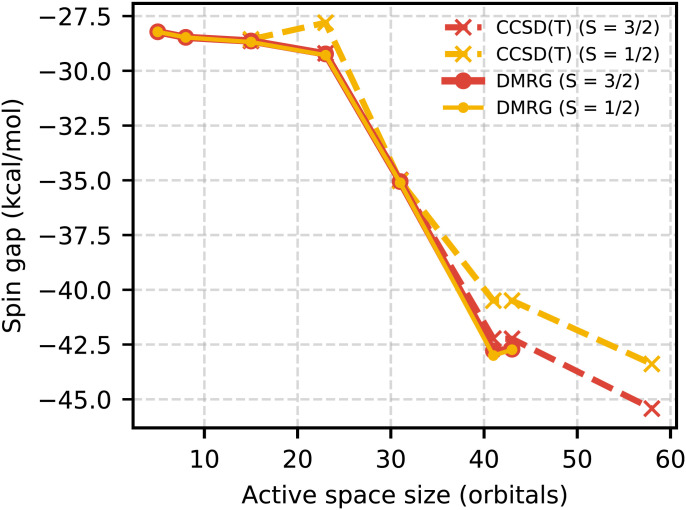
Energy differences between the high-spin sextet (S = 5/2) and low-spin doublet (S = 1/2) and quartet (S = 3/2) states of Cpd I. Only for the doublet is there significant disagreement between CCSD(T) and DMRG energies within the active space. DMRG consistently predicts the doublet and quartet states to be nearly degenerate.

**Fig. 3.**
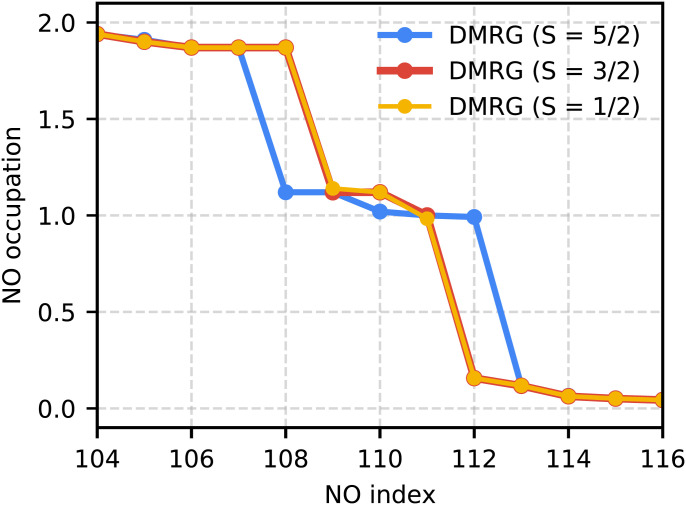
Natural orbital occupation numbers from the DMRG density matrix in the 41-orbital “G” active space for the sextet (S = 5/2), quartet (S = 3/2), and doublet (S = 1/2) states. Note that the doublet clearly has three singly occupied orbitals.

In all active spaces considered for Cpd I, we found the sextet to be much higher in energy than the nearly degenerate doublet and quartet states. This is largely consistent with previous calculations (25, 33, 34). In Fig. 2 we show the DMRG and CCSD(T) spin gaps within the specified active space. CCSD(T) and DMRG agree to within 0.1 kcal/mol for the sextet and quartet states indicating single-reference character. However, for the doublet state, the DMRG energy is consistently much lower than the CCSD(T) energy. The DMRG natural orbital (NO) occupation numbers shown in Fig. 3 reveal that the doublet state has three open-shell natural orbitals. This state is not well described in traditional coupled-cluster theory, and accurate computation therefore requires a method, such as DMRG, that is capable of treating systems with multiconfigurational character.

**Fig. 4.**
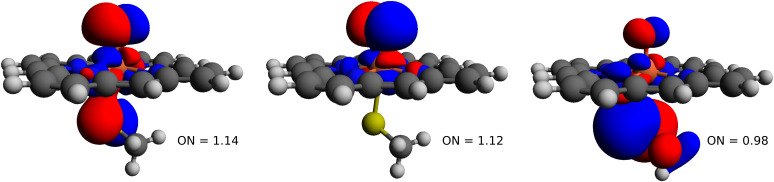
Visualization of the three singly occupied natural orbitals of the doublet state of Cpd I.

Our DMRG results for the G active space are the largest DMRG calculations yet performed on any model of Cpd I. We find the doublet state to be lower than the quartet by only 0.02 kcal/mol. Thus, from these estimates both the doublet and quartet states would be expected to be populated at room temperature. The sextet is 42.7 kcal/mol higher in energy than the two low-spin states. This is qualitatively consistent with most past calculations on this system (9, 25, 33, 34, 53). We show the three singly occupied NOs in Fig. 4, along with their occupation numbers (ONs), from which we can see that there are two Fe-O orbitals with $\pi_{xz}^{*}$ and $\pi_{yz}^{*}$ character and a third sulfur nonbonding orbital. In this case the sulfur nonbonding orbital is mixed with one of the Fe-O $\pi^{*}$ orbitals. This is consistent with studies that use S-Me or the full cysteine ligand in the gas phase (9, 53). In studies that either use S-H in place of the cysteine ligand (32, 46) or include the protein environment in some way (54, 55), the third orbital is found to be mixed with a heme *a_u_* ($\pi^{*}$) orbital.

The near degeneracy of the doublet and quartet states makes prediction of the lowest-energy spin state very difficult for this system. In particular, including the remaining dynamic correlation from outside of the active space can change this picture qualitatively (see the results in *SI Appendix*, section 5). While NEVPT2 can provide a good estimate of the relatively large dynamic correlation energy, it is not accurate enough to resolve the doublet and quartet states. In this system, the addition of the NEVPT2 correlation energy can even shift the relative energy of the sextet such that it becomes the lowest spin state in some of the active spaces. Without larger, prohibitively expensive calculations, one cannot distinguish a real effect from an artifact of the various approximations. Furthermore, details of the protein model, dielectric environment, and cysteine ligand model can easily shift the energies by enough to change the qualitative result (56). Together, these sources of uncertainty indicate that reliable identification of the lowest spin state of Cpd I as it appears in most experiments is not feasible. However, unambiguous identification of the lowest spin state in our model compound could be possible with a DMRG calculation in a very large active space, such as our active space, followed by a NEVPT2 correction for the remaining dynamic correlation. As the active space is made larger, the NEVPT2 correction becomes smaller and more reliable, although a DMRG-NEVPT2 calculation of this size is beyond our current capability. It is interesting to note that in a system like this, a very large active space is required not because there are a large number of strongly correlated orbitals, but rather because this is the only well-known, reliable means to obtain a balanced combination of static and dynamic correlation for different spin states.

### Cost Estimates for Classical Computation.

The computational cost of DMRG calculations depends on the bond dimension, *M*, and number of active orbitals, *k*. The bond dimension, *M*, is an adjustable parameter that controls the quality of the calculation, and convergence of the energy with respect to *M* must be carefully monitored to ensure accurate results. The theoretical asymptotic scaling of computational resources required for a DMRG calculation with a given bond dimension and number of active orbitals has been discussed elsewhere (57). The scaling of the central processing unit (CPU) time is $O(k^{3}M^{3})$, and the memory and disk requirements scale as $O(k^{2}M^{2})$ and $O(k^{3}M^{2})$, respectively. Given the theoretical scaling, we can estimate the computational cost of a calculation in the 58-orbital X active space. These estimates are shown in Table 1. For the StackBlock program, which implements both shared-memory and distributed-memory parallelism, it may be possible to perform an M=3,000 calculation on the 58-orbital X active space with a significant investment of computational resources over a period of approximately 1 mo of wall time. However, there is no guarantee that a bond dimension of M=3,000 will be sufficient and it is likely that calculations of even higher bond dimension are required.

**Table 1.** Actual resources required for DMRG calculations in the 43-orbital G active space and estimated resources needed for DMRG calculations in the 58-orbital X active space

	G (M=1,500)	X (M=1,500)	X (M=3,000)
CPU time, h	1,800	4,570	36,564
Memory, GB	48	87	348
Disk, GB	235	572	2,288

For systems like our model of Cpd I that do not have a quasi–one-dimensional structure the bond dimension required to converge a DMRG calculation to the required precision will grow in such a way to make the overall scaling of the method weakly exponential. In practice, this means that active spaces larger than the X active space described here quickly become intractable.

## 2. Quantum Computing Resource Estimates

In this section we perform a detailed accounting of the space and time complexity for sampling from the eigenbasis of the active-space Hamiltonians within the context of error-corrected quantum computers. The space resources we consider are the total number of logical qubits and physical qubits required to perform the phase estimation algorithm. For time complexity we focus on the number of Toffoli gates that are the rate-limiting gate operation within the surface code error correction protocol (58). To provide a range of estimates we consider various error rates of the qubits composing the surface code and optimize success probability for the full quantum algorithm and code distance. We demonstrate assuming physical qubit error rates of 0.1% that the ground-state energy of the largest model Hamiltonian for the active-space X of Cpd I can be assessed with ~4.6 million physical qubits in 73 h of runtime. Anticipating improvements in qubit technology and reduction of error rates to 0.001% the same computation preparing the ground state of Cpd I can be performed in ~500,000 physical qubits and 25 h of runtime.

The prevailing method for using a quantum computer to learn about the eigenspectrum of a molecular system is through phase estimation. The quantum computer simulates an operator related to the chemical system and learns spectral information without having to sacrifice accuracy beyond the basis set errors inherent to specifying the Hamiltonian. In terms of the electronic structure Hamiltonian (*H*) the phase estimation algorithm implements unitaries generated by a simple function of the Hamiltonian *f*(*H*), which, when applied to a state ψ, accumulates phases according to the spectrum of *f*(*H*) (59):

[1] $$U|\psi \rangle =\sum_{k}\langle k|\psi \rangle e^{-if(E_{k})}|k\rangle ,$$

where *k* indexes an eigenstate of *f*(*H*). Leveraging the quantum Fourier transform, a phase can be approximately determined to error *ϵ* by applying *U* to the state $\frac{1}{\epsilon }||\partial f(E)/\partial E|{|}^{-1}$ times and then performing a projective measurement. In this scheme, the probability of measuring the ground state of *f*(*H*) to error *ϵ* is $|\langle 0|\psi \rangle {|}^{2}$. Therefore, the parameters controlling the cost of phase estimation are the error *ϵ*, the cost of implementing *U*, and the overlap with the ground state $|\langle 0|\psi \rangle {|}^{2}$. We assess all these factors for CYP active spaces to obtain leading-order estimates of the space complexity and time complexity (Toffoli counts) for obtaining the ground-state energy with the phase estimation protocol.

There are numerous variations of phase estimation that all come with different cost models depending on the type of Hamiltonian function, *f*(*H*), and the cost of implementing *U*. For example, the function that expresses a Hamiltonian as a sum of Hermitian operators $f(H)=\sum_{l}H_{l}$ would result in a simple unitary under Trotterization whose spectrum is close to the original Hamiltonian *H*. Recent work (20, 60, 61) has shown that applying phase estimation to the qubitization iterate (62) allows one to learn eigenvalues and prepare eigenstates with error no greater than *ϵ* by repeated application of the relevant quantum operator—the so-called qubitized quantum walk operator— O(λ/ϵ) (63) times where *λ* is the L1-norm of the coefficients of the Hamiltonian. *λ* is associated with the cost of implementing the qubitization walk operator *f*(*H*). The resulting function *f*(*H*) turns out to be proportional to the arccosine of *H* and thus the unitary *U* is an operator whose eigenvalues are $e^{\pm i\text{arccos}(E)}$, where *E* are eigenvalues of the original Hamiltonian (20, 64).

Three previous papers performed a full resource estimation on the number of physical qubits and runtime requirements for simulating chemical systems in a molecular orbital basis using various forms of the qubitized quantum walk protocol (20, 60, 61). A detailed description of their differences, costs, and implementations can be found in ref. 64 but here we highlight the differences relevant to quantum chemistry. Most generically, for all qubitization schemes the walk operators can be implemented in Toffoli complexity scaling as $O(\sqrt{\Gamma })$ and $O(\sqrt{\Gamma })$ ancilla qubits, where Γ is the amount of information needed to specify a particular tensor factorization of the Hamiltonian coefficients where each tensor factorization can be implemented in O(N) Toffoli complexity. Generally, the Hamiltonian coefficients we consider are the scalars associated with the two-electron Coulomb repulsion integrals. Using these costs, phase estimation can be implemented in a total gate complexity of $\tilde{O}(\sqrt{\Gamma }\lambda /\epsilon )$.* In all methods Γ and *λ* are nontrivially related to each other through the particulars of tensor factorization of the Hamiltonian coefficients. Thus, to find the best schemes for chemical systems we study the performance of three instances of qubitization schemes using different tensor factorizations in full detail to assess the overall scaling of each technique.

The three Hamiltonian factorization schemes we compare are the single factorization (SF) (60, 65) (related to the Cholesky decomposition of the two-body electronic structure Hamiltonian) (67), double factorization (DF) (61, 65, 67), and tensor hypercontraction (THC) (68–70) with Γ costs articulated in Table 2. In both the DF and THC factorizations each tensor factor is evolved by rotating into a basis such that the central tensor is diagonal. This rotation costs *O*(*N*) Givens rotations (61), which yield linear Toffoli complexity. For THC the basis rotation is a projection into a larger basis with rank equal to the THC rank. In prior work utilizing the THC decomposition on the two-electron integral tensor within the qubitization framework, a brute-force optimization scheme was used to determine the THC decomposition involving random restarts and direct gradient descent on the least-squares objective (20). In this work we use a variation of prior THC decomposition workflows by starting with a symmetric canonical polyadic decomposition of the Cholesky vectors followed by L1-regularized optimization of the THC factors. Full details of this protocol are described in *SI Appendix*, section 7. We validate that this scheme reliably produces small *λ* values for increasing system size.

**Table 2.** Tabulation of space and time complexity of the cost of performing phase estimation on qubitized quantum walk operators

Tensor	Space	
factorization	complexity	Toffoli
method	(logical qubits)	complexity
SF	$\tilde{O}(N^{3/2})$	$\tilde{O}(N^{3/2}\lambda /\epsilon )$
DF	$\tilde{O}(N\sqrt{\Xi })$	$\tilde{O}(N\sqrt{\Xi }\lambda /\epsilon )$
THC	$\tilde{O}(N)$	$\tilde{O}(N\lambda /\epsilon )$

Generically, qubitized quantum walks scale as $\tilde{O}(\sqrt{\Gamma })$ in space and $\tilde{O}(\lambda \sqrt{\Gamma }/\epsilon )$, where Γ is the amount of information required to specify the Hamiltonian within a particular tensor factorization. For the SF method $\Gamma =\tilde{O}(N^{3})$. For double factorization $\Gamma =\tilde{O}(N^{2}\Xi )$, where Ξ is the average rank of the second factorization that is expected to scale as *O*(*N*) in most regimes (65). For THC $\Gamma =\tilde{O}(N^{2})$, assuming the THC rank grows linearly with system size.

### Quantum Resource Scaling.

**Fig. 5.**
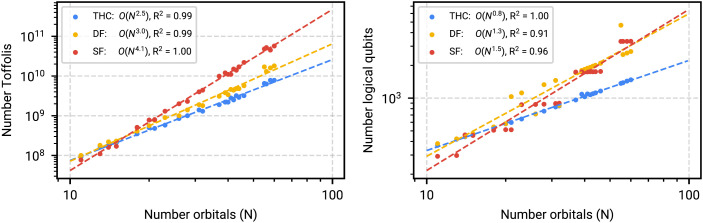
(Left) Number of Toffolis as a function of the number of active-space orbitals for the four heme compounds in this study, grouped by factorization algorithm: THC (blue), DF (yellow), and SF (red). Empirical scaling of the number of Toffolis as a function of orbitals N is obtained by a least-squares fit on the log-log plot. (Right) Number of logical qubits as a function of the number of active-space orbitals for the four heme compounds in this study, grouped by factorization algorithm: THC (blue), DF (yellow), and SF (red). Empirical scaling of the number of qubits as a function of orbitals N is obtained by a least-squares fit on the log-log plot.

Each factorization scheme requires a user-specified cutoff in terms of how accurately the two-electron integral tensor (and thus the underlying Hamiltonian) should be represented. This cutoff directly affects the scaling of the algorithms. To efficiently estimate the cutoff for a fixed accuracy we use CCSD(T) with two-electron integrals reconstructed with a specific cutoff for the high-spin electron configurations. As validated previously, CCSD(T) is accurate in the active space for all compounds with a high-spin electron configuration. For example, to determine the sufficiently accurate THC rank, we perform THC decompositions with increasing rank until the CCSD(T) error is within one milliHartree (~0.6 kcal/mol). One milliHartree is selected for consistency with previous work (20) but it may be necessary to select an even lower cutoff due to the fact that errors are additive between the total success probability of phase estimation, rounding in the procedure implementing the oracles associated with qubitization via Quantum Read Only Memory (QROM) rounding, and truncation of two-electron integrals via approximate factorization. From here, the *λ* for each factorization is computed and subsequently input into the detailed cost estimates for each step of the walk operator construction. Ref. 20 contains a full description of all costs associated with optimal implementation of each of the oracles in the qubitized quantum walk operator. For reproducibility we provide a software tool that takes as input various tensor factorizations and computes the total resource requirements in terms of logical qubits and Toffoli counts. The process of determining the THC rank cutoff for the largest active space of Cpd I is shown in Table 2. To estimate a generic THC rank to avoid the process of rank optimization we aggregate all determined THC ranks for all four compounds in all active spaces and perform a linear regression. The generic THC rank is determined to be 4.7 (see *SI Appendix*, section 7, Fig. 7 for details). As previously described, the true scaling of qubitization for each factorization depends largely on how well the various factorizations can use the existing structure in the two-electron integral tensors and compress the information. Using the progressively larger active spaces and resource estimates in terms of Toffoli gate and logical qubit counts for each factorization, we can assess the scaling and extrapolate to larger size factorizations not studied in this work. The extrapolations for number of Toffoli gates and number of logical qubits required are shown in Fig. 5.

### Compilation into Surface Code.

To perform the required number of gates for phase estimation the quantum state must be protected against errors through a quantum error correction protocol. The surface code is one such protocol that can be implemented on a two-dimensional array of qubits and requires physical error rates no worse than 0.5% (71). In the surface code one can make trade-offs between space and time, i.e., changing the length of time required for the computation at the expense of using more physical qubits. Significant controlling factors for this are the number of Toffoli factories and how these resource factories are implemented.

We start with analyzing the runtime requirements for simulating the largest X Hamiltonians. For these systems dynamic correlation corrections may be small and are thus more likely to result in accurate spin gaps. To estimate the cost of executing phase estimation in the surface code, we start from the number of data qubits and Toffoli gates required, determined from the previous section based on a THC factorization of the two-electron integrals. We assume that four magic-state factories are being used and the execution time is determined by the Toffoli count, due to being bottlenecked waiting for magic-state factories. Higher factory counts would require a much different analysis on routing overheads. We assume space usage is determined by the number of logical data qubits, the number of magic-state factories, and a 50% overhead for routing. Finally, we assume a physical per-gate error rate of 0.1%, a surface code cycle time of 1 ms, and a control system reaction time of 10 ms.

These assumptions are used to determine a variety of different configurations (code distances and factory layouts) and to select the configuration that uses the least spacetime volume while ensuring that the quantum computation corrects all errors at least 90% of the time. The code to do this estimation is provided as a submodule of OpenFermion (72). For physical qubit error rates below 0.1% we use self-correcting CCZ (AutoCCZ) factories from ref. 58 and T factories from ref. 73. To get an overall failure rate for the entire algorithm for a given configuration we estimate the failure rate of the factories and the failure rate of logical data qubits.

**Fig. 6.**
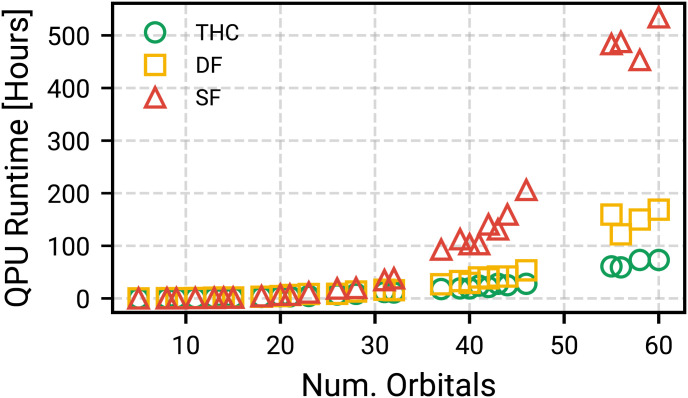
Phase estimation runtimes with physical qubit error rate of 0.1% and four Toffoli factories while assuming a 1-µs surface code cycle. While the THC factorization has substantial leading-order costs to account for, over the simpler to implement single factorization, the asymptotic advantage of THC compression is demonstrated at small system sizes.

**Fig. 7.**
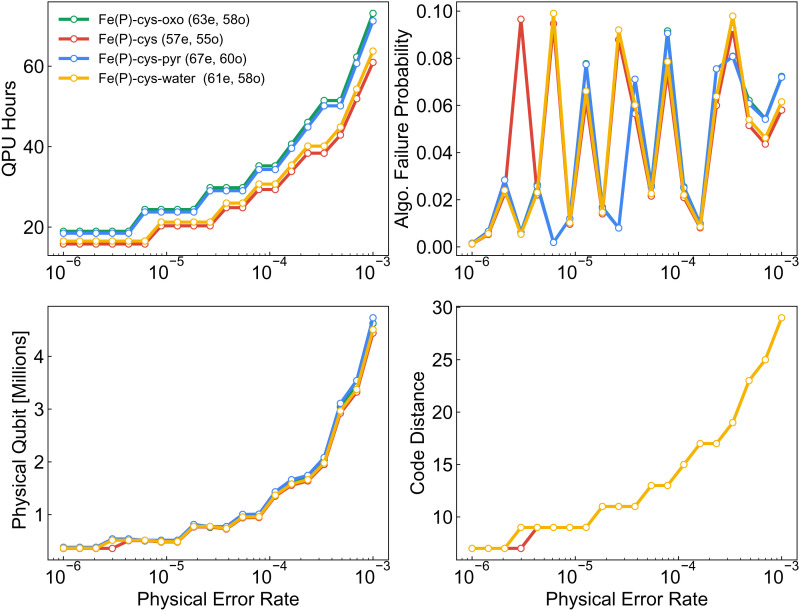
Comparison of compiled resource requirements as a function of physical error rate. (Top Left) Runtime. (Top Right) Algorithm failure probability. (Bottom Left) Physical qubit requirements. (Bottom Right) Required code distance. We note that error rates on the order of $1.0\times 10^{-6}$ are highly unrealistic and if achieved would precipitate using a different error correction protocol.

For a physical error rate of 0.1%, four factories, and the aforementioned surface-code timings the optimal configuration was AutoCCZ magic-state factories with a level-1 code distance of 19 and level-2 code distance of 31 and logical data qubits with a code distance of 29. This requires 4,624,440 physical qubits and a total runtime of 73 h. For these estimates we assume that the space–time requirements (qubits and Toffolis) are translated directly into error-corrected requirements, which is an overestimation of the needed resources. Thus these estimates should be viewed as upper bounds assuming other timing criteria are met by real machines. In Fig. 6 we plot the runtime scaling for phase estimation performed on all active-space calculations with different tensor factorizations. The data show that despite higher prefactors for tensor hypercontraction qubitization the asymptotic scaling advantage appears even at small sizes. As a forecasting exercise we can make runtime, physical qubit, and code distance estimates as a function of physical qubit error rate. This provides insight into the scenario of qubit error rates becoming substantially better. We again make the assumption that surface code cycle and reaction times are 1 µs and 10 µs and we use four Toffoli factories. In Fig. 7 we show the decreased runtime, physical qubit requirements, and code distances as a function of physical error rate. In the event we have extremely low gate error rates of 0.001%, solving the largest system will take only 500,000 qubits and 25 h of runtime.

### Demarcating the Quantum Advantage Boundary.

**Fig. 8.**
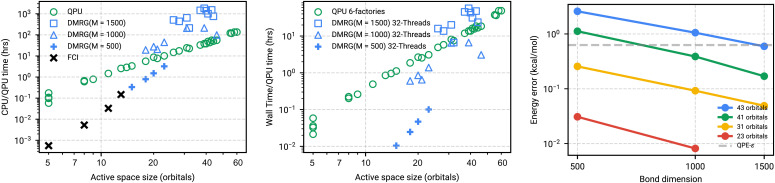
(Left) CPU time for Complete Active Space Configuration Interaction (CASCI) CASCI/DMRG calculations on different active spaces and QPU time to perform phase estimation on the THC-decomposed active-space Hamiltonian. Times are determined by wall clock time multiplied by the number of threads. QPU time is determined assuming a 0.1% gate error rate and two Toffoli factories and neglects repetitions needed due to potentially small initial-state overlap. Both methods, save CASCI, are parallelizable to some extent by using more resources. (Center) Same timings as Right but now considering 32 threads for DMRG and six Toffoli factories for the QPU. (Right) DMRG energy at fixed bond dimension relative to extrapolated energy for various numbers of orbitals.

Using timings from DMRG calculations and estimated runtimes on an error-corrected quantum computer we can compare the corresponding CPU and quantum processing unit (QPU) time requirements as a function of active-space size to investigate the potential for a simulation advantage. In Fig. 8 we plot the measured timings of DMRG calculations on all active spaces for different values of bond dimension (*M*) and the estimated runtimes for the quantum computing to perform phase estimation. The QPU runtimes are estimated using two Toffoli factories, which corresponds to 4.9 million qubits and 135 h of runtime for the largest systems. DMRG runtimes are computed as the wall clock time multiplied by the number of threads. In practice the runtime of either calculation can be reduced by using more cores or more Toffoli factories, respectively. To put the DMRG timings in context relative to QPU timings we need a notion of how accurate they are at fixed bond dimension. Due to the lack of convergence in any of the DMRG or coupled-cluster calculations for the spin gap, we instead compare to the extrapolated DMRG energy for a given active space. This comparison makes the assumption that the extrapolated energy at any bond dimension is highly accurate. Fig. 8 suggests that for large bond (*M* ≥ 1,000) dimension and large active space quantum phase estimation already has a computational advantage when the task is simulating the ground-state energy.

While DMRG provides access to reduced-density matrices (and therefore other observables), phase estimation provides only estimates of the energy and further processing is required to compute other quantities. This is important for energetic quantities as well, because corrections for dynamic correlation outside of the active space usually require reduced-density matrices (1-, 2-, 3-, and sometimes 4-particle). These corrections, such as the NEVPT2 used in this work, can allow for smaller active-space calculations in principle. This difference highlights a need for quantum algorithms addressing dynamic correlation. Extrapolating out the number of qubits and Toffoli gates required for CYP active-space models at 500 orbitals (which is approximately the number of orbitals used for the full-space coupled-cluster calculations) would require ~9,000 logical qubits and 1.5 trillion Toffoli gates to perform phase estimation on the entire space. Thus, the development of quantum algorithms for addressing dynamic correlation is an important step toward chemical computational relevancy.

**Fig. 9.**
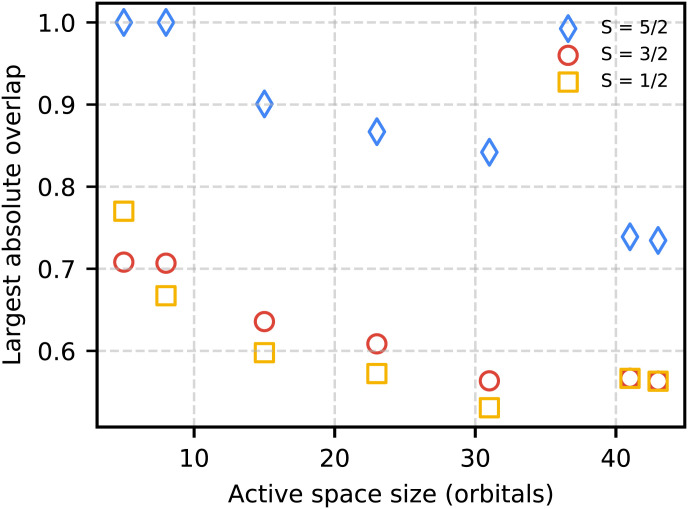
Largest computational basis-state overlap with the ground-state DMRG M=1,500 wavefunction.

The success probability for phase estimation also relies on overlap *S* of the initial state with an eigenstate of the Hamiltonian. Therefore, a full timing comparison would ideally factor the overlap dependency as a $\mathrm{poly}(S^{-1})$ multiplying prefactor that can be improved with knowledge of the gap (74). To have an idea of the size of this prefactor for Cpd I we have computed the determinant used in the exact diagonalization basis, also known as a computational basis state, that has the largest overlap with the DMRG M=1,500 bond dimension wavefunction for systems A through G. These overlaps were computed using the block2 program (75). In Fig. 9 we plot the overlap as a function of active-space size for all three spin states considered. As expected, the largest overlap decays by a factor of 2 going from a system size of 10 qubits to 84 qubits but never falls to a value that would be problematic for phase estimation.

## Conclusion

To demarcate the quantum advantage boundary for biologically relevant compounds we have performed a detailed characterization of the classical and quantum resources required to accurately describe the electronic structure of the P450 enzyme active site. This work highlights the demand of careful application of a variety of classical chemical methods and that even in regimes where a problem is not “strongly correlated” quantum computers can potentially provide advantage due to the need to treat dynamic and static correlation in a balanced way. We also determine that circumventing the traditional strategy of partitioning dynamic and static correlation by using a quantum computer to simulate the entire problem is unlikely to be feasible due to the very large number of resource states needed to execute phase estimation. These findings suggest that further development of fault-tolerant algorithms, either in observable extraction or in scaling, are necessary for quantum computers to be transformative for simulating electronic structure where modest multireference character is augmented by a large dynamic amount of correlation.

To make the aforementioned conclusions we analyzed the multireference character through a variety of classical electronic structure methods that help to clearly determine the computational frontier of CYP simulation. The necessity of using multireference methods is supported by examining spin contamination, defined by $\langle S^{2}-S_{z}^{2}-S_{z}\rangle$, as well as three other metrics from correlated wavefunction theory, namely, max($\left|t_{1}\right|$) and max($\left|t_{2}\right|$) from CCSD, as well as natural orbital occupation numbers from regularized κ-OOMP2 (Regularized Orbital-Optimized Second-Order Møller-Plesset Perturbation Theory [kappa-OOMP2]). From these metrics, we found that Cpd I displayed some multireference character. DMRG calculations confirm the triradical character involving three open shells in the Cpd I doublet corresponding to Fe-O $\pi_{xz/yz}^{*}$ orbitals and a lone pair orbital on the sulfur atom of the Me-S ligand emulating the full coordinating cysteine ligand. This triradical character is consistent with previous multireference calculations on Cpd I (33, 55, 76). Despite a clear characterization of nontrivial open-shell electronic structure in Cpd I, the ground-state spin state and spin gaps remain elusive due to the need to treat dynamic and static correlation on equal footing.

Analysis of the quantum resources required to simulate these systems indicated that of the three Hamiltonian factorizations (SF, DF, and THC factorization) used in qubitized phase estimation, tensor hypercontraction consistently outperformed the other two. Compilation into the surface code provided an upper-bound runtime estimate for executing phase estimation. Most notably, under realistic hardware configurations we predict that the largest models of CYP can be simulated with under 100 h of quantum computer time using approximately 5 million qubits implementing $7.8\times 10^{9}$ Toffoli gates using four T factories. A direct runtime comparison of qubitized phase estimation shows a more favorable scaling than DMRG, in terms of bond dimension, and indicates future devices can potentially outperform classical machines when computing ground-state energies. Extrapolating the observed resource estimates to the full Cpd I system and compiling to the surface code indicate that a direct simulation of the entire system could require 1.5 trillion Toffoli gates—an unfeasible number of Toffoli gates to perform.

The classical benchmarking of CYP compounds demonstrates the need to account for dynamic correlation and the quantum cost estimates detailing the requirements for a high-accuracy simulation encourage further development of quantum algorithms to address multireference quantum chemistry beyond the strong correlation regime. Furthermore, this work demonstrates that classical calculations play an important role in guiding quantum algorithms research and are essential for defining the computational frontier for chemistry.

## Supplementary Material

Supplementary File

## Data Availability

For reproducibility, we share data and code used in this work on a public Zenodo repository (10.5281/zenodo.5941130) (78), including the molecular geometries and active-space Hamiltonians, along with the inputs to reproduce the calculations. All software used for Hamiltonian tensor factorizations and phase estimation resource estimates can be found in the resource_estimates module of OpenFermion (72) commit no. cf53c063d0f124a02ff8776bb7f8afb110d4bde6. To perform the tensor hypercontraction factorization with the code in OpenFermion an interface, pybtas, to BTAS (77) commit 5702259d5d207fe5a8e0c975c3cf1f610dcf381a is required. The pybtas library is included in the Zenodo repository and can be found at https://github.com/ncrubin/pybtas (79). The THC factors obtained from this work are also available in the Zenodo repository.

## Footnotes

^*^The $\tilde{O}(.)$ indicates asymptotic scaling but dropping any parameters that scale as polynomials of logarithms in that parameter.
