# Supplementary material for: Reliably assessing the electronic structure of cytochrome P450 on today’s classical computers and tomorrow’s quantum computers
Source: Proc Natl Acad Sci U S A. 2022 Sep 12;119(38):e2203533119. doi: 10.1073/pnas.2203533119 (PMC9499570; doi:10.1073/pnas.2203533119)
Supplement: Supplementary File [file pnas.2203533119.sapp.pdf]

Supplementary Information: Reliably assessing the electronic structure of cytochrome P450 on today's classical computers and tomorrow's quantum computers

Joshua J. Going[a,1], Alec White[b,1], Joonho Lee[a,c], Christofer S. Tautermann[d,e], Matthias Degroote[f], Craig Gidney[a], Toru Shiozaki[b], Ryan Babbush[a,2], and Nicholas C. Rubin[a,2]

[a]Google Quantum AI, Venice, CA 90291, United States; [b]Quantum Simulation Technologies, Inc., Boston, 02135, United States; [c]Department of Chemistry, Columbia University, New York, New York 10027, USA; [d]Boehringer Ingelheim Pharma GmbH & Co KG, Birkendorfer Strasse 65, 88397 Biberach, Germany; [e]Department of General, Inorganic and Theoretical Chemistry, University of Innsbruck, 6020 Innsbruck, Austria; [f]Quantum Lab, Boehringer Ingelheim, 55218 Ingelheim am Rhein, Germany

This manuscript was compiled on August 1, 2022

1. Electron correlation and the computational cost of electronic structure calculations

The nature of the electronic correlation in a particular system influences the choice of appropriate electronic structure method. For systems with wave functions that are dominated by a single electronic configuration—that is, single reference—it is generally sufficient to treat the electronic correlation using low-order perturbation theory, for example, with the hierarchy of coupled cluster methods. These methods quantitatively correct for the lack of electron correlation by incorporating excited Slater determinants into the wave function. These excited Slater determinants generally yield small contributions, in the sense that that their wave function amplitudes are relatively small compared to the dominant zeroth-order reference. That said, the number of excited determinants that need to be considered may be large. When a large number of small corrections to the wave function is required to treat electron correlation, the correlation is often referred to as "dynamic correlation." Dynamic correlation is straightforward (though not necessarily cheap!) to systematically improve, simply by increasing the order of theory.

In contrast, for systems where no single dominant configuration is sufficient to describe the wave function even qualitatively, low orders of perturbation theory will fail. Several electronic determinants are required, and their contribution to the overall wave function will be of roughly equal magnitude. Such a system is considered "multireference" or "strongly correlated," and the electron correlation is often referred to as "static correlation" "Static" and "dynamic" correlation can be viewed as two classification extremes that are useful for developing methods. For many systems, careful consideration of both types of electronic correlations are required, and demand methods that can balance accuracy of describing "static" and "dynamic" correlation. As both are required to treat chemical systems with sufficient accuracy, it is important to not treat one type of correlation more accurately than the other: insufficient accuracy in one will lead to errors computed properties, regardless of how accurate the other correlations were computed. A balanced treatment of electron correlation is not a trivial task and imbalances may lead to difficulties in describing the spin-state ordering, as we will show in the model systems discussed in this work.

Generally, it is not possible to know *a priori* whether or not a chemical system is either single or multireference. Though several heuristics have been established (e.g. does the system contain multiple metal sites?), the only reliable way to determine if a problem is multireference is to subject it to calculation. This presents a quandary: on the one hand, one wants to avoid expensive multireference methods where possible, yet on the other hand, the most reliable way to determine if such methods are necessary is to employ said methods. This, coupled with the fact that there is no clear single "measure" of strong correlation, makes scoping the problem very difficult. To this end, several probes of strong correlation have been proposed that, when taken together, give one reasonable confidence that a multireference approach is or is not necessary.

In this work, we examine the merits of four commonly used probes, or diagnostics, of strong correlation:

1. Spin symmetry breaking. It is generally thought that spin-symmetry broken wave functions indicate the presence of strong correlation, for example, beyond the Coulson-Fisher point in homolytic bond dissociation. This can be quantified by computing the spin-contamination, defined as the $\langle S^2 - S_z^2 - S_z \rangle$ expectation value. However, the use of spin-contamination as a diagnostic of strong correlation is not as clear cut. Recent work (1) has shown that spin-symmetry broken mean-field solutions may be "cleaned up" by either by switching to a different SCF reference (such as one based on Kohn-Sham DFT) or by utilizing an orbital-optimized method in the presence of dynamic correlation, such as κ-OOMP2 (2). References that can have their spin symmetry "restored" in this manner are referred to as "artifically" spin-symmetry broken solutions. Those that cannot, on the other hand, show "essential" spin-symmetry breaking and do indeed indicate some measure of multireference character or strong correlation. As an example, prior work on the bare iron porphyrin complex details extensive calculation providing evidence that the complex is single reference (3) and arguing that spin-contamination is largely a product of "artificial" symmetry breaking from not including an accurate treatment of dynamic correlation at the single-determinant level of

[1] contributed equally to this work.

[2]To whom correspondence should be addressed. E-mail: nickrubin@google.com, babbush@google.com

DRAFT

theory.

2. Measures based on the coupled cluster singles amplitudes. While norms of the CCSD t_1 amplitudes, such as the T_1 or D_1 diagnostic (4–6), are commonly used to evaluate whether a system is strongly correlated, these measures are more of a measure of the quality of the underlying orbitals. The reason is that in coupled cluster theory, the single excitations act as an orbital rotation operator. In many cases, choosing a more suitable reference is sufficient to eliminate large t_1 norms. One additional problem with relying on norms of the t_1 amplitudes is that it masks heterogeneity in the singles amplitudes, i.e. if only a small number of singles amplitudes are large, the norm will not be representative. For this reason, we report the $\max(|t_1|)$ value, as has been done elsewhere (7–10).

3. Measures based on the coupled cluster doubles amplitudes. In contrast to the singles, measures based on the doubles amplitudes have a clear connection to electron correlation, as they generate configurations that cannot be obtained by merely rotating the reference wave function. Moreover, doubles amplitudes only have a weak dependence on the underlying reference. Here report the $\max(|t_2|)$ value (7–13). When this value is large (e.g. $\max(|t_2|) > 0.1$) this is a reliable indication the system is multireference. (Recall that due to intermediate normalization in coupled cluster theory, the reference amplitude is always unity.)

4. Natural orbital occupation numbers from correlated methods. Natural orbital occupation numbers (NOONs), which are the eigenvalues of the one-particle reduced-density matrix (1PDM), can deviate from the idealized open-shell character when using correlated 1PDMs. In this case, the NOONs indicate that additional open shells are present (e.g. polyradicaloid character) which indicates unambiguously that the chemical system is multireference. In this work, we consider NOONs from the κ-OOMP2 method, which provides an affordable route to evaluating multireference character.

Although this list is by no means exhaustive, it does present some commonly utilized diagnostics used by the electronic structure community to evaluate multireference character.

2. Cytochrome P450

The superfamily of cytochrome P450 (CYPs) contains membrane-bound heme containing enzymes which function mostly as monooxygenases. In the human genome 57 CYP isoforms are encoded, moreover, it is the largest family of hemoproteins known, with probably more than 300,000 members across all organisms (14). CYPs also catalyze other reactions like dealkylations, dehalogenations, and epoxidations (15). Still, the oxidation function is most important, being involved in the biosynthesis of steroids, hormones, as well as fatty acids (16). Therefore, the major role of CYPs, which is the task they are known for, lies in the detoxification of organisms. The most common detoxification mechanism, displayed in the catalytic cycle in Figure 1, involves a single oxygen insertion into C-H bonds of CYP substrates, thereby generating a hydroxy-group, which enables the further processing (like glucuronidation) or enhances direct excretion due to higher hydrophilicity of the metabolites. The oxidation by CYPs is also the most common metabolization mechanism for drugs in humans, where more than 70% of all drugs are metabolized by just two CYP isoforms (CYP 3A4 and CYP 2D6) (17). Therefore, the interaction of CYPs and small organic molecules is of high interest in drug design. Structurally the CYPs contain a buried heme-iron center, where the iron is bound to a highly conserved cysteine thiolate side chain of the protein. The secondary and tertiary structure has been quite conserved through evolution (18) and especially the cysteine-facing amino acid environment close to the heme is highly conserved in CYPs. However, on the distal side of the heme group channels towards the solvent allow the entry of small organic molecules to the CYP active site above the heme group. In the case of CYP 3A4, which is known to roughly metabolize 50% of all marketed drugs (19), also more than one substrate molecule can be accommodated in the active site(20).

Many drugs are known to inhibit some CYP isoforms (21), and the potential problem of inhibitors lies in the potential of drug-drug interactions. The reason is that inhibition of drug metabolism results in reduced degradation of potential other substrates of that CYP isoform. In the worst case this leads to drug accumulation and higher drug levels in the body, increasing the risk for potential side effects (22). A variety of inhibitor bound CYP structures is known, and a recurring structural motif is the presence of aromatic nitrogen atoms that are directly coordinated to the iron center of the heme group (23). In Figure 2 we show a selection of inhibiting drug bound structures of CYP 3A4, where nitrogen arene substrucures of the inhibitors directly coordinate to the heme iron. CYP inhibiting substructures include triazole (contained in fluconazole, antifungal drug - pdb: 6MA7 (24, 25)), pyridine (metyrapone, drug against adrenal insufficiency - pdb: 6MA6 (25, 26)), oxazole (desoxyritonavir analogue - pdb: 4I4G (27, 28)), imidazole (ketoconazole, antifungal - pdb: 2V0M (29, 30)) and thiazole (ritonavir, co-medication to HIV treatment - pdb: 3NXU (31, 32)). From Figure 2 a clear interaction pattern can be deduced, as the nitrogen atom of the heteroarene structures directly binds to the iron center as 6th coordination partner in addition to the porphyrine nitrogens and the thiolate. Inhibitors, therefore often directly interact with the iron and the interaction strength contributes strongly to the overall potency of an inhibitor. Therefore a proper understanding of the molecular actions of small ligands on P450 enzymes as well as an understanding of the catalytic mechanism is of central importance for drug design. The heme group with its iron center, however, causes difficulties in the theoretical description of the relevant complexes, due to strong dependence on the protein environment and the high degree of electron correlation in the catalytic center. Therefore, to model substrate oxidation or ligand specificity, the protein and membrane environment have to be taken into account.(33, 34) That means that multiscale approaches have to be employed,(35, 36) where the active site is described by QM/MM methods and the environment is modeled by force fields.

.1. Geometries. The geometries of the model systems were derived from experimental X-ray structures of CYP3A4 and are displayed in Figure 1 of the main text. The PDB access codes for the structures are 1TQN and 6MA6 for the water- and pyridine-bound complexes respectively. The model systems

DRAFT

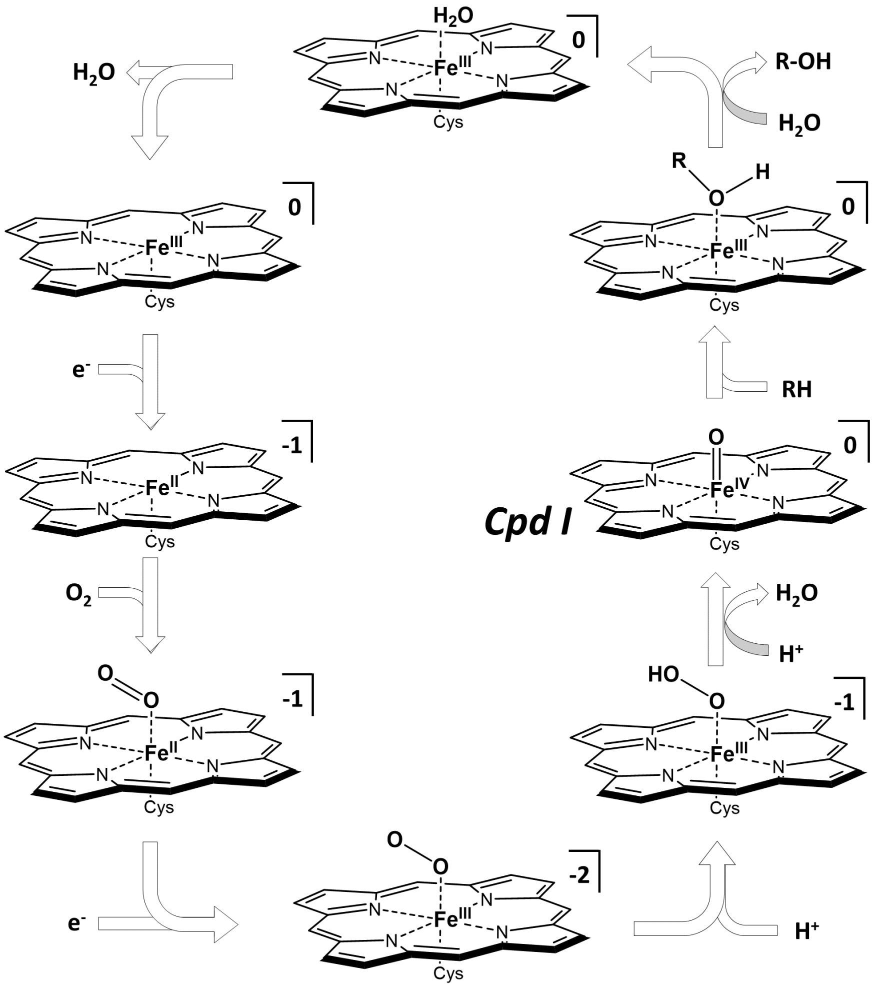

Fig. 1. Catalytic cycle of P450 to oxidize aliphatic R-H bonds: Starting with water and Cys-thiolate bound heme (top) as resting state, water dissociates, and subsequently an electron is added to the system yield a 5-valent Fe(II) complex. Now molecular oxygen is associating to Fe(II) and stepwise addition of an electron and two protons cleaves the O-O bond to release one water entity. The remaining species is Compound I, where an oxygen atom is bound to Fe (formally in oxidation state IV). Compound I is the reactive species, and the addition of an aliphatic system (here denoted by RH) leads to an insertion of the oxygen into the R-H bond. The formed alcohol R-OH dissociates and water takes its place, yielding the resting state again and thereby closing the catalytic cycle.

were prepared from the crystal structure by removal of all non-iron coordinating entities of the protein and the solvent. The iron-coordinating cysteine is truncated to methyl-thiolate and all porphyrine-attached substituents have been removed. The coordinating 6th ligand of the iron was truncated at the pyridine in the case of 6MA6. The pentacoordinated state has been modeled by removing the water from the 1TQN model system. Cpd I models were derived from the water bound model system by removing both water bound hydrogens. The prepared geometries were optimized by UB3LYP (37–40) with the cc-pVDZ basis set (41) employing LANL2DZ pseudopotentials (42–44) on the iron center by Gaussian09 (45) and standard convergence criteria. To improve convergence, the virtual orbitals were shifted by 0.1 Hartree. All model systems were of neutral charge and the overall spin multiplicity was set to 2 and 6 for low and high spin complexes respectively.

3. Computational details for full space and active space calculations

.1. Full valence space calculations. To estimate the degree to which these compounds are strongly correlated, κ-OOMP2 and CCSD calculations were performed on these systems. Although these methods are single-reference methods, they were chosen to provide a reasonable estimate of the total electronic structure, as well as provide probes (such as the $\max(|t_1|)$ and $\max(|t_2|)$ diagnostics from CCSD) for evaluating potential multireference character. A complete discussion of the multireference character metrics used in this work can be found in Appendix 1. The κ-OOMP2 calculations were performed in the full electronic space, while for efficiency CCSD utilized the common frozen-core approximation. These calculations were performed using the Q-Chem 5.4 electronic structure package (46). A value of $\kappa = 1.1$ was used for the κ-OOMP2 method, as recommended for transition metal complexes (47). The geometry for each molecule corresponded to the B3LYP high-spin (S=5/2) optimized geometry. All calculations used the cc-pVDZ (41) basis with the RI approximation. To test

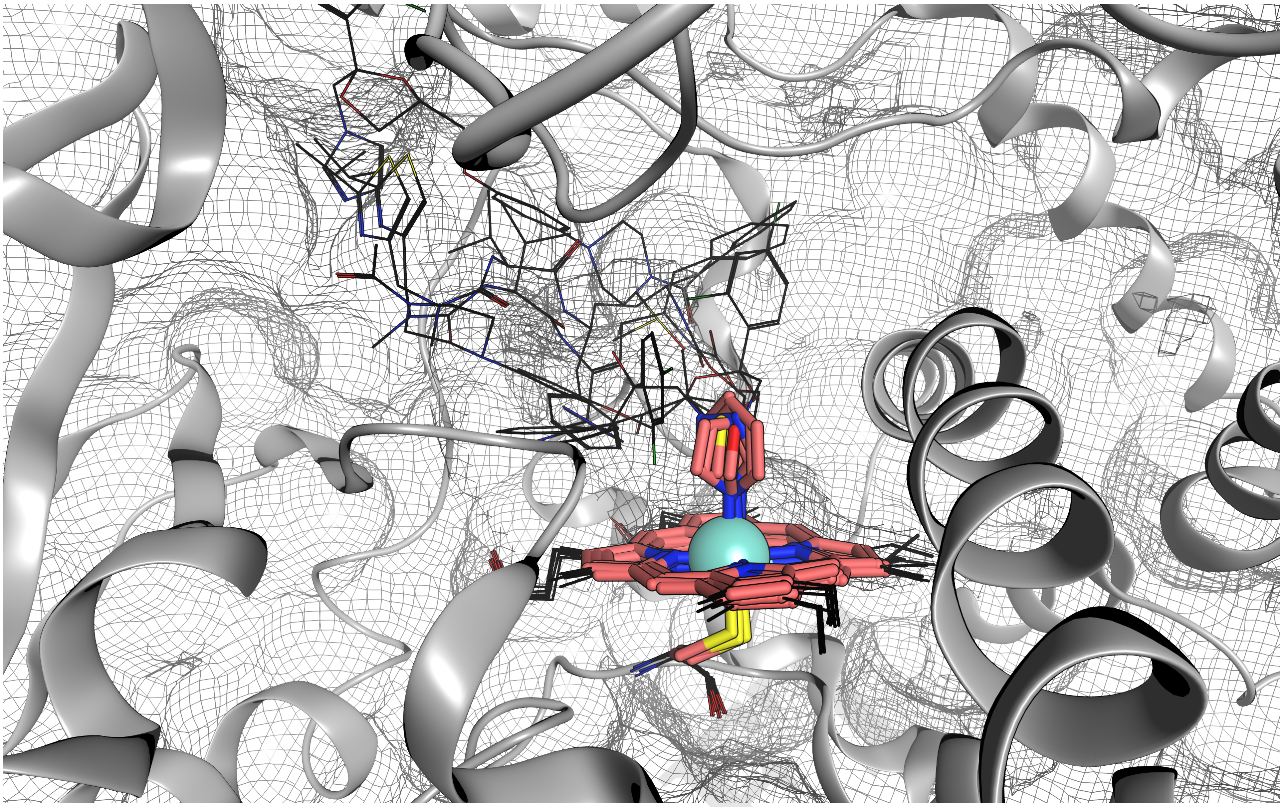

Fig. 2. Overlay of various CYP 3A4 structures bound to N-arene containing inhibitors - pdb structures 6MA7, 6MA6, 4I4G, 2V0M and 3NXU are displayed. Heme, thiolate, heteroarene-substructure of inhibiting drugs are displayed in salmon cylinder representation, the rest of the inhibitor structures as well as the thiolate bearing cysteine are shown as black line representations, and the rest of the CYP 3A4 protein is drawn as cartoon backbone and mesh surface in grey.

the impact of reference orbitals, CCSD calculations were performed using UHF, UB3LYP (37–40), and UBP86 (48, 49) orbitals. B3LYP and BP86 functionals were chosen to compare with a hybrid and a GGA functional, and the two functionals are among the most commonly used in chemistry. All references corresponded to stable, local minima. The orbitals obtained from KS-DFT were semi-canonicalized prior to the CCSD calculations. To evaluate the presence of multireference character in these compounds, the spin contamination of the reference $\langle S^2 - S_z^2 - S_z \rangle$, as well as the the max($|t_1|$) and the max($|t_2|$) diagnostics from CCSD were evaluated, where t_1 and t_2 are the CCSD amplitudes. From the κ-OOMP2 calculations, the natural orbital occupation numbers (NOONs) were computed by diagonalizing the corresponding one-particle density matrix (1PDM) in order to observe deviation from the idealized open-shell character (1).

A. Active space calculations. To evaluate the quality of a given active space for fault-tolerant calculations on a quantum computer, DMRG calculations were performed on each active space for the doublet, quartet, and sextet multiplicities. DMRG calculations were performed with StackBlock (Block 1.5.0) (50) via the PySCF (51, 52) interface. The reported energies are extrapolated based on the discarded weight computed from a reverse sweep schedule as described by Olivares-Amaya *et al* (53). N-electron valence state perturbation theory (NEVPT2) (54) calculations as described by Sharma *et al.* (55) were used to compute an estimate of dynamic correlation effects.

In addition to the DMRG calculations, CCSD with non-iterative triples (CCSD(T)) (56) calculations were performed on the active space models with varying spin states. The coupled cluster calculations were performed using the PySCF (51, 52) unrestricted coupled cluster code starting from the localized orbitals obtained from restricted open-shell Hartree-Fock, as detailed above. To account for changes in spin multiplicity, the orbitals were re-optimized within the active space prior to the CCSD(T) calculations.

4. Details of DMRG calculations on Cpd I

Some of the details of the DMRG calculations on our model of Cpd I are shown in Table 1. The extrapolation error is estimated by dividing the difference between the largest calculation and the extrapolated value by 5. This empirical rule has been used in the past to provide a rough estimate of the extrapolation error (53).

We also note that, for the purposes of resource estimation, the expected asymptotic scaling of $O(k^3)$ for k active orbitals and a fixed bond dimension is empirically observed for the system sizes considered here (See Fig. 3).

Table 1. Number of orbitals, max bond dimension, and extrapolation error for the DMRG calculations on Cpd I. Note that calculations on active spaces A-C where performed exactly, and the X active space was too large for practical calculations.

name	# of orbitals	Max bond dimension	Extrapolation error-d,q,s (kcal/mol)
B	8	-	-
C	15	-	-
D	23	1000	2E-3, 2E-3, 7E-5
E	31	1500	1E-2, 1E-2, 6E-3
F	41	1500	3E-2, 6E-3, 2E-3
G	43	1500	1E-1, 2E-1, 1E-1
X	58	-	-

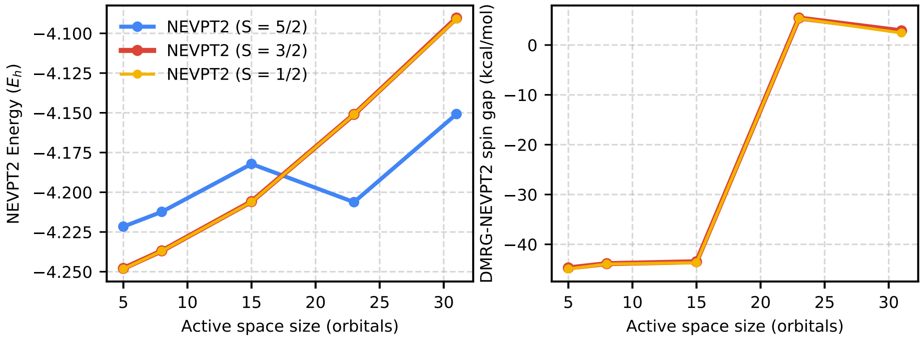

Fig. 4. *left*: NEVPT2 energies, and, *right*: DMRG-NEVPT2 spin gaps in active spaces A-E. In the D and E active spaces, the addition of the NEVPT2 correlation energy dramatically changes the spin ordering so that the high-spin state is lowest in energy.

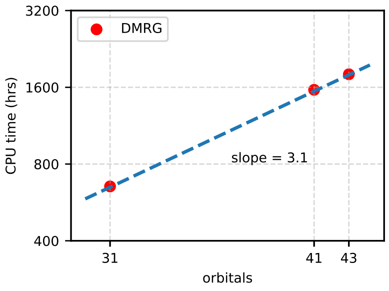

Fig. 3. CPU time for DMRG calculations of fixed bond dimension ($M = 1500$) as a function of the active space size. The data are plotted on a log-log scale, and the slope of trendline, ~ 3.1 in this case, provides an estimate of the scaling for this fixed bond dimension. In practice, the high scaling of DMRG calculations comes from the increase in the necessary bond dimension with system size.

5. DMRG+NEVPT2 calculations on Cpd I

In Figure 4 we plot the NEVPT2 correction to each spin state for CASCI/DMRG wavefunctions in some of the smaller active spaces. In the D and E active spaces, small differences in the NEVPT2 correlation energy are nonetheless large enough to qualitatively change the spin ordering. However, this could be an artifact the NEVPT2 approximation itself, the approximate NEVPT2 implementation used in this work (55), or the approximate DMRG active-space wavefunction.

6. Numerical support for a multireference treatment of p450

The electronic structure of CYPs has been extensively studied computationally in the literature using both polynomial-scaling methods such as DFT (15) and using active space methods (57–59). Although DFT-based simulations have successfully revealed many aspects of potential reaction mechanisms (15), it has been argued that there is a need for application of active space based methods to achieve high accuracy for spectroscopic properties (57) and spin-state orderings (58–60). Our cost assessment for simulation on classical and quantum hardware in the previous sections has been based on this assumption. In the following, we will provide further numerical evidence that predictive simulation of the catalytic cycle of CYPs requires a multireference treatment. We investigate the multiconfigurational character of the model compounds with full-space (no active space) calculations by considering four commonly-used diagnostics: single-determinant spin contamination, $\max(|t_1|)$ and $\max(|t_2|)$ diagnostics from CCSD (7–13), and deviations from ideal spin multiplicity as given by natural orbital occupation numbers from the κ-OOMP2(1, 61) method. For completeness we provide a detailed discussion on the chemical meaning of each metric in Appendix 1.

First we examine the $\max(|t_1|)$ diagnostic from CCSD which characterizes the *quality* of reference orbitals (7–10). This quantity is plotted in Figure 5 for orbitals obtained from two flavors of Kohn-Sham density functional theory and Hartree-Fock theory. With Hartree-Fock orbitals, all compounds have a relatively large $\max(|t_1|) > 0.19$, suggesting that Hartree-Fock is a poor reference for these systems. However, using Kohn-Sham orbitals, which include dynamic correlation effects, is sufficient to yield a significantly lower value of $\max(|t_1|)$ for all compounds except for the iron-oxo containing Cpd I. This shows that for three of the four compounds (empty pentacoordinate, resting, and pyridine inhibited) the relatively large $\max(|t_1|)$ can be corrected by including some dynamic correlation in the reference orbitals. In other words, with an improved SCF reference, the systems appear to be single-reference. However, this is not the case for Cpd I given the KS orbitals considered here, which suggests that a single-

DRAFT

reference approach may struggle to capture the true nature of electron correlation.

Another commonly used diagnostic of multiconfigurational character is spin-symmetry breaking in the wave function (9, 11), seen in Figure 5. The reference orbitals given here are from unrestricted calculations, and may deviate from ideal eigenstates of S^2, shown by the nonzero expectation value of spin contamination, $\langle S^2 - S_z^2 - S_z \rangle$. Like large values of $\max(|t_1|)$, spin contamination is often used to identify underlying multiconfigurational character. For example, it has been used to support the claim the C_{60} is polyradical (62), despite experimental evidence to the contrary. While spin contamination can, in some cases, be a genuine marker of multiconfigurational character (so-called "essential" symmetry breaking), it may also be the result of "artificial" symmetry breaking, due to the lack of dynamic correlation. We observe that for Hartree-Fock orbitals, the spin contamination is large for all compounds and all spin states investigated. However, upon switching the reference orbitals to Kohn-Sham orbitals, we note that the purported spin contamination is essentially eliminated, barring some spin contamination in the doublet of Cpd I. This indicates, once again, that by carefully choosing an appropriate SCF reference (here with KS orbitals) it is possible to properly describe the system with single reference methods. In most cases, DFT greatly reduces large $\max(|t_1|)$ values as well as eliminates spin contamination, showing the spin contamination observed in the HF references as "artificial". The exception is the doublet state of Cpd I.

A more rigorously justified diagnostic of strong correlation (or multiconfigurational character) is the $\max(|t_2|)$ diagnostic from CCSD (7–13). Large amplitudes from the double excitations, generally considered when $\max(|t_2|) > 0.05 - 0.1$, are relatively insensitive to the underlying reference orbitals. Unlike single excitations in CCSD, which are essentially orbital rotations, doubles amplitudes directly capture the lowest-order pairwise correlations between electrons. We observe that for all compounds and all spin states, the value of $\max(|t_2|)$ is more-or-less constant, roughly centered around $\max(|t_2|) \approx 0.05$. Depending on the threshold for $\max(|t_2|)$ (we would consider the more stringent and common $\max(|t_2|) > 0.1$ to be a marker of strong correlation), the data show that none of the various model compounds are particularly strongly correlated. In light of the other diagnostics and the fact that using KS orbitals appears to restore most compounds to a reasonable single-reference configuration, we see no evidence that the P450 model compounds are strongly correlated.

Finally, we examine the natural orbital occupation numbers (NOONs) from the correlated 1PDMs of κ-OOMP2 calculations as shown in Figure 6. For the three compounds that we expect to have largely single-reference character, the sextet, quartet, and doublet states have 5, 3, and 1 open-shells respectively as expected. However, the "doublet" state of Cpd I has 3 NOONs close to one indicating a state of triradical character which is consistent with prior calculations (59). Though the $\max(|t_2|)$ diagnostic does not indicate that the Cpd I doublet is any more strongly correlated than the other model compounds, such a state cannot be faithfully represented by a single determinant. Though one can compute some properties, such as the nuclear gradient, with symmetry-broken states, it is often difficult to accurately compute spin-dependent properties, like the spin gap, without spin-pure states.

Taken together, we, like others, conclude that P450 appears to have a multiconfigurational electronic structure. A treatment of electron correlation that can approach the exact solution is essential to resolve the nearly degenerate quartet and doublet states. In other words, to achieve the high accuracy needed to assess spin energetics and other properties, multireference methods are required. Doing this reliably is not an easy task. Though Cpd I is not "strongly correlated" in the sense that it does not have a large number of strongly entangled orbitals, there is no easy way to do reliable simulation, and the need for multireference methods transcends the set of strongly correlated problems. This motivates the need to develop quantum algorithms and methods that can scale to sizes large enough to balance dynamic and static electron correlation.

7. Tensor Hypercontraction by Optimized CP3

The Tensor Hypercontraction (THC) technique (63–65) has emerged as one of the most performant strategies for implementing qubitization based simulation strategies within fault tolerant quantum computing (66). The THC decomposition is used in quantum chemistry to decompose the two-electron integral tensor from a four-tensor into five smaller tensors. Mathematically, the THC is

$$(ij|kl) = \sum_{PQ}^{M} X_{iP} X_{iQ} Z_{PQ} X_{kQ} X_{lQ} \qquad [1]$$

where the THC rank M scales as $M = \mathcal{O}(N \mathrm{poly}\log(1/\epsilon_{\mathrm{THC}}))$ where ϵ_{THC} is the energy error due to the factorization per atom as we scale to the basis set limit (67). The THC factorization is related to the canonical polyadic (CP) decomposition (68, 69) but cannot be obtained through simple linear algebra relations. One strategy to obtain the THC factors is through the CP decomposition of geminal terms through alternating least-squares (68) with an iteration complexity of $\mathcal{O}(r^4 M)$ where r is the size of the single particle basis. Extensive numerical studies suggest the rank of M should scale linearly in the basis set size r with a small integer prefactor (usually 3-6). This has been benchmarked across a wide variety of chemical applications. Our interest in the THC factorization is that the cost of the repeated circuit primitives for the qubitized quantum walk oracles SELECT and PREPARE can be directly related to the L_1-norm of the central tensor Z_{PQ}. Thus our goal is not only to perform a high accuracy THC decomposition on the two-electron integral four-tensor but also minimize the norm of the central tensor. The norm minimization is not directly taken into consideration through standard THC decomposition techniques introduced in the quantum chemistry field.

Following Ref. (66) we start with an initial guess for the tensors X_{iP} and Z_{PQ} and optimize the L_2 difference loss function

$$L(X,Z) = \sum_{ijk} \left|(ij|kl) - \sum_{PQ}^{M} X_{iP} X_{iQ} Z_{PQ} X_{kQ} X_{lQ}\right|^2 \qquad [2]$$

$$+C \sum_{PQ} |Z_{PQ}| \qquad [3]$$

by L-BFGS-B implemented in Scipy. Due to the non-linear nature of the objective function the quality of the initial starting guess can drastically change the likelihood of finding a

DRAFT

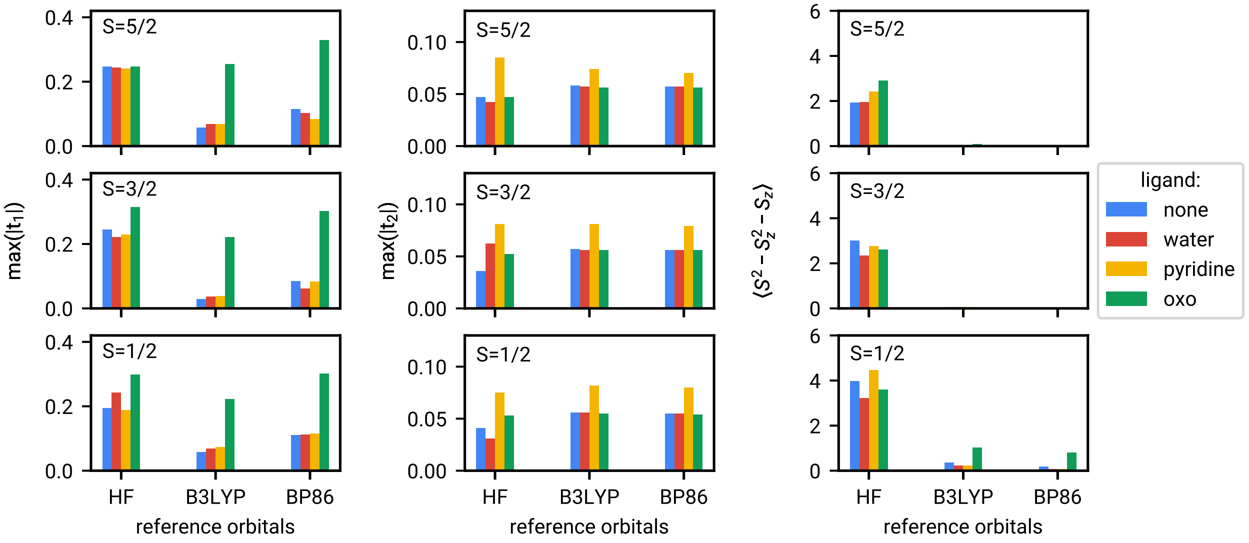

Fig. 5. *left*: Impact of reference orbital on the $\max(|t_1|)$ values from CCSD. *center*: Impact of reference orbital on the $\max(|t_2|)$ values from CCSD. *right*: Spin contamination, $\langle S^2 - S_z^2 - S_z\rangle$, as a function of reference orbitals. For most species, use of KS reference orbitals removes nearly all of the spin contamination in the SCF, in contrast to the highly-spin contaminated HF orbitals. The doublet (S=1/2) and quartet (S=3/2) states for the oxo ligand in Cpd I remain somewhat spin contaminated despite using KS reference orbitals.

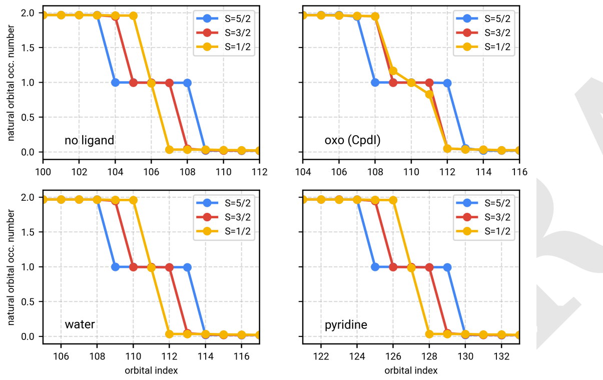

Fig. 6. κ-OOMP2 ($\kappa = 1.1$) natural orbital occupation numbers for the four compounds and three associated spin states. All calculations are performed at the high-spin (S=5/2) geometry obtained from a B3LYP geometry optimization, as detailed in the text. The open-shell character of all compounds corresponds to the idealized spin multiplicity, with the lone exception being the iron-oxo Cpd I doublet, which shows three open shells for the doublet state, in agreement with the DMRG calculations in this work as well as previous calculations indicating triradical character, such as in Ref. (59).

stationary point corresponding to a factorization that minimizes the L_2 loss and has a small L_1-norm for the central tensor. To find an initial X and Z we use the symmetric CP decomposition, denoted CP3, on the Cholesky factors, B, of the 2-electron integral tensor

$$(ij|kl) = \sum_{\chi} B_{ij,\chi} B_{kl,\chi} = \mathbf{B}\mathbf{B}^T \qquad [4]$$

where $\mathbf{B}$ is obtained either from an SVD or improved scaling algorithms (70). Forming a symmetric decomposition of B

$$B_{ij,\chi} = \sum_{\tau} \beta_{i,\tau}\beta_{j,\tau}\zeta_{\chi,\tau} \qquad [5]$$

is accomplished with alternating least-squares implemented in the C++ Basic Tensor Algebra Subroutines (BTAS) library (71). A Python implementation of the cost function and gradients for Eq. Eq. (2) was used to optimize the THC tensors for all systems we considered. The L_1 regularizer C in Eq. Eq. (2) was set such that the L_2 difference had the same strength as the L_1 part of the loss function given an initial set of THC factors from CP3. We found that without regularization of the L_1 norm THC decomposition from CP3 followed by L-BFGS-B optimization can provide erratically large $|Z_{PQ}|$ leading to erratic fault tolerant cost estimates for simulation of the systems considered here. As a consistency check we ensure that as the THC rank M is increased the L_2 norm of the difference between the true tensor and THC reconstructed two-electron integral tensor decreases along with a smooth increase in $\sum_{PQ}|Z_{PQ}|$. The smooth convergence of λ, CCSD(T) error, and logical qubit count for different size THC factors is shown in Table 2.

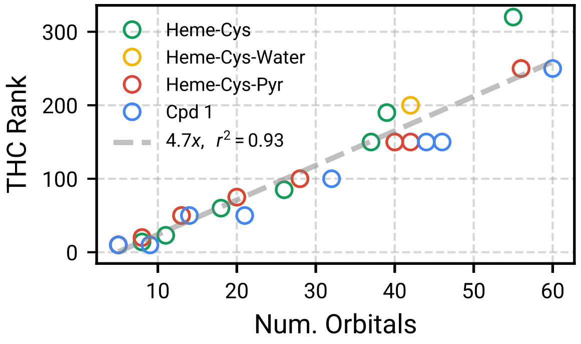

Fig. 7. Number of orbitals versus the THC rank for active spaces A through X for all four compounds.

To minimize pre-computation for quantum algorithms it is

DRAFT

Table 2. Large active space Cpd I using CAS((34α, 29β), 58o) and showing the convergence of CCSD(T) for the high spin Hamiltonian as a function of the THC rank M. 320 is the largest THC rank we consider for resource estimates.

M	||ERI - THC||	CCSD(T) error (mEh)	lambda	Toffoli count (10^9)	logical qubits
140	4.7531	−107.80	260.4	4.3	1426
160	2.4689	−28.33	326.7	5.6	1426
180	2.3111	−12.62	331.0	5.8	1426
200	1.5772	−5.27	352.3	6.3	1429
220	0.46249	−0.84	385.0	7.0	1429
240	0.70397	−1.45	377.6	7.0	1429
260	0.52769	−0.20	382.8	7.3	1434
280	0.45739	−1.29	384.8	7.4	1434
300	0.36635	−1.17	387.4	7.6	1434
320	0.31506	0.10	388.9	7.8	1434
340	0.24618	−0.14	390.9	8.0	1434
360	0.22478	−0.71	391.6	8.2	2156
380	0.19167	−0.83	392.5	8.3	2158
400	0.15139	−0.45	393.7	8.5	2158

important to select the correct rank M for THC without having to search. In Figure 7 we plot the THC rank obtained from searching for a CCSD(T) error less than 1 milliHartree with respect to the full two-electron integral tensor. We observed that the THC rank is approximately 5 times the size of the one-particle basis used in the problem which agrees well with previous chemistry studies.

8. Active space sizes

Starting from the high spin ROHF orbitals localized with the Pipek-Mezey scheme a class of active spaces is selected for each compound in the high spin configuration. The logic for generating the active space hierarchy is to identify the orbitals most relevant to the ligand binding and spin character, expanding outward in terms of relevance and/or spatial localization, all the while adding same character virtual orbitals as needed to maintain a constant (roughly 50%) filling fraction. The process is as follows: the open-shells are expected to be most important for spin-character so the five open-shells are selected for the first active space. After the Fe d-orbitals are expected to be most important so the remaining occupied 3d orbitals are selected to yield active space ‘B’ along with Iron 4s and (spatially) nearby iron-nitrogen anti-bonding orbitals to yield a roughly 50% filling fraction. Subsequently, axial ligand (anti-)bonding orbitals were added to this set to yield active space ‘C’ along with Iron orbitals mixing 4p and d’ orbitals. These axial orbitals were selected as they are expected to be the most relevant to both the ligand binding as well as the overall spin state character. Finally, we augment the active spaces by expanding to orbitals spatially outward from this “core active space” of metal and axial metal-ligand orbitals. Iron-nitrogen sigma orbitals are spatially closest, and adding these along with their corresponding virtuals yield active space ‘D’. The heme π orbitals are expected to stabilize the metal core, so we next added the heme π orbitals from the nitrogen, as these bond directly to the metal (active space ‘E’). Next, we expand to the heme π orbitals from the carbon atoms (active space ‘F’). Finally, the active spaces were augmented with the more distal carbon-sulfur (anti-)bonding orbitals (active space ‘G’) and then the less-important heme carbon-nitrogen sigma orbitals (active space ‘X’). The same set of orbitals were used for active space calculations at different spin-states.

1. J Lee, M Head-Gordon, Distinguishing artificial and essential symmetry breaking in a single determinant: Approach and application to the c 60, c 36, and c 20 fullerenes. *Phys. Chem. Chem. Phys.* **21**, 4763–4778 (2019).
2. J Lee, M Head-Gordon, Regularized Orbital-Optimized Second-Order Møller-Plesset Perturbation Theory: A Reliable Fifth-Order-Scaling Electron Correlation Model with Orbital Energy Dependent Regularizers. *J. Chem. Theory Comput.* **14**, 5203–5219 (2018).
3. J Lee, FD Malone, MA Morales, Utilizing essential symmetry breaking in auxiliary-field quantum monte carlo: Application to the spin gaps of the c36 fullerene and an iron porphyrin model complex. *J. Chem. Theory Comput.* **16**, 3019–3027 (2020).
4. TJ Lee, PR Taylor, A diagnostic for determining the quality of single-reference electron correlation methods. *Int. J. Quantum Chem.* **36**, 199–207 (1989).
5. ML Leininger, IMB Nielsen, TD Crawford, CL Janssen, A new diagnostic for open-shell coupled-cluster theory. *Chem. Phys. Lett.* **328**, 431–436 (2000).
6. TJ Lee, Comparison of the T1 and D1 diagnostics for electronic structure theory: a new definition for the open-shell D1 diagnostic. *Chem. Phys. Lett.* **372**, 362–367 (2003).
7. CJ Cramer, M Włoch, P Piecuch, C Puzzarini, L Gagliardi, Theoretical models on the Cu2O2 torture track: mechanistic implications for oxytyrosinase and small-molecule analogues. *J. Phys. Chem. A* **110**, 1991–2004 (2006).
8. DG Liakos, F Neese, Interplay of correlation and relativistic effects in correlated calculations on transition-metal complexes: The (Cu2O2)(2+) core revisited. *J. Chem. Theory Comput.* **7**, 1511–1523 (2011).
9. W Jiang, NJ DeYonker, AK Wilson, Multireference character for 3d Transition-Metal-Containing molecules. *J. Chem. Theory Comput.* **8**, 460–468 (2012).
10. L Cheng, J Gauss, B Ruscic, PB Armentrout, JF Stanton, Bond dissociation energies for diatomic molecules containing 3d transition metals: Benchmark Scalar-Relativistic Coupled-Cluster calculations for 20 molecules. *J. Chem. Theory Comput.* **13**, 1044–1056 (2017).
11. M Radon, Spin-state energetics of heme-related models from dft and coupled cluster calculations. *J. Chem. Theory Comput.* **10**, 2306–2321 (2014).
12. JT Margraf, A Perera, JJ Lutz, RJ Bartlett, Single-reference coupled cluster theory for multi-reference problems. *The J. Chem. Phys.* **147**, 184101 (2017).
13. M Feldt, QM Phung, K Pierloot, RA Mata, JN Harvey, Limits of Coupled-Cluster calculations for Non-Heme iron complexes. *J. Chem. Theory Comput.* **15**, 922–937 (2019).
14. DR Nelson, Cytochrome p450 diversity in the tree of life. *Biochimica et Biophys. Acta (BBA) - Proteins Proteomics* **1866**, 141–154 (2018).
15. S Shaik, et al., P450 enzymes: Their structure, reactivity, and selectivity—modeled by qm/mm calculations. *Chem. Rev.* **110**, 949–1017 (2010).
16. AA Hajeyah, WJ Griffiths, Y Wang, AJ Finch, VB O'Donnell, The biosynthesis of enzymatically oxidized lipids. *Front. Endocrinol.* **11**, 910 (2020).
17. FP Guengerich, Cytochrome p450 and chemical toxicology. *Chem. Res. Toxicol.* **21**, 70–83 (2008).
18. EF Johnson, CD Stout, Structural diversity of eukaryotic membrane cytochrome p450s*. *J. Biol. Chem.* **288**, 17082–17090 (2013).
19. YT Liu, HP Hao, CX Liu, GJ Wang, HG Xie, Drugs as cyp3a probes, inducers, and inhibitors. *Drug Metab. Rev.* **39**, 699–721 (2007).
20. A Boobis, et al., Drug interactions. *Drug Metab. Rev.* **41**, 486–527 (2009).
21. J Hakkola, J Hukkanen, M Turpeinen, O Pelkonen, Inhibition and induction of cyp enzymes in humans: an update. *Arch. Toxicol.* **94**, 3671–3722 (2020).
22. SN Rao Gajula, MS Pillai, G Samanthula, R Sonti, Cytochrome p450 enzymes: a review on drug metabolizing enzyme inhibition studies in drug discovery and development. *Bioanalysis* **13**, 1355–1378 (2021).
23. MB Shah, J Pascual, Q Zhang, CD Stout, JR Halpert, Structures of cytochrome p450 2b6 bound to 4-benzylpyridine and 4-(4-nitrobenzyl)pyridine: Insight into inhibitor binding and rearrangement of active site side chains. *Mol. Pharmacol.* **80**, 1047–1055 (2011).
24. IF Sevrioukova, Human cyp3a4 bound to an inhibitor fluconazole (2019).
25. I Sevrioukova, Interaction of human drug-metabolizing cyp3a4 with small inhibitory molecules. *Biochemistry* **58**, 930–939 (2019).
26. IF Sevrioukova, Human cyp3a4 bound to an inhibitor metyrapone (2019).
27. IF Sevrioukova, TL Poulos, Crystal structure of cyp3a4 ligated to oxazole-substituted desoxyritonavir (2013).
28. IF Sevrioukova, TL Poulos, Pyridine-substituted desoxyritonavir is a more potent inhibitor of cytochrome p450 3a4 than ritonavir. *J. Medicinal Chem.* **56**, 3733–3741 (2013).
29. T Sjogren, M Ekroos, Crystal structure of human p450 3a4 in complex with ketoconazole (2007).
30. M Ekroos, T Sjögren, Structural basis for ligand promiscuity in cytochrome p450 3a4. *Proc. Natl. Acad. Sci.* **103**, 13682–13687 (2006).
31. IF Sevrioukova, TL Poulos, Crystal structure of human cytochrome p4503a4 bound to an inhibitor ritonavir (2010).
32. IF Sevrioukova, TL Poulos, Structure and mechanism of the complex between cytochrome

DRAFT

Table 3. A summary of our strategy for constructing the hierarchy of active spaces used in this work. Each active space is formed by adding the listed orbitals to the active space on the preceding line beginning with the ‘A’ active space consisting of only the singly occupied orbitals. We show the size of the active space, in number of orbitals, for our model of Cpd I in the final column.

name	occupied orbitals	virtual orbitals	size (Cpd I)
A	Singly-occupied orbitals	-	5
B	Iron 3d	Iron 4s/iron-nitrogen anti-bonding	8
C	Iron-axial ligand(s) bonding	Iron 4p/d'/iron-axial ligand anti-bonding	15
D	Heme iron-nitrogen bonding	Iron 4p/d'/iron-nitrogen anti-bonding	23
E	Heme π bonding (localized around N atoms)	Heme π anti-bonding (localized around N atoms)	31
F	Heme π bonding (localized around C atoms)	Heme π anti-bonding (localized around C atoms)	41
G	Cysteine carbon-sulfur bonding	Cysteine carbon-sulfur anti-bonding	43
X	Heme carbon-nitrogen bonding	Heme carbon-nitrogen anti-bonding	58

Table 4. Active space systems with number of orbitals and number of electrons in the active space. ‘Rest’ corresponds to the resting state of the CYP active site which involves a water molecule as the sixth coordination to the cys-Fe(P) complex. ‘Empty’ corresponds to the pentacoordinate cys-Fe(P) system with no sixth ligand. ‘Cpd1’ is compound 1 and inhibited is the cys-Fe(P) system with a pyridine inhibitor coordinate to the sixth ligand site.

Active Space	rest		empty		inhibited		Cpd1	
	orb.	elec.	orb.	elec.	orb.	elec.	orb.	elec.
A	5	5	5	5	5	5	5	5
B	8	9	8	9	9	11	8	11
C	13	15	11	13	9	17	15	19
D	20	23	18	21	21	25	23	25
E	28	31	26	29	32	35	31	33
F	40	43	37	39	44	49	41	45
G	42	45	39	41	46	51	43	47
X	56	61	55	57	60	67	58	63

DRAFT

p4503a4 and ritonavir. *Proc. Natl. Acad. Sci.* **107**, 18422–18427 (2010).

33. F Ogliaro, S Cohen, SP de Visser, S Shaik, Medium polarization and hydrogen bonding effects on compound i of cytochrome p450: What kind of a radical is it really? *J. Am. Chem. Soc.* **122**, 12892–12893 (2000).
34. R Lonsdale, J Oláh, AJ Mulholland, JN Harvey, Does compound i vary significantly between isoforms of cytochrome p450? *J. Am. Chem. Soc.* **133**, 15464–15474 (2011).
35. R Lonsdale, et al., Quantum mechanics/molecular mechanics modeling of regioselectivity of drug metabolism in cytochrome p450 2c9. *J. Am. Chem. Soc.* **135**, 8001–8015 (2013).
36. R Lonsdale, SL Rouse, MSP Sansom, AJ Mulholland, A multiscale approach to modelling drug metabolism by membrane-bound cytochrome p450 enzymes. *PLOS Comput. Biol.* **10**, 1–16 (2014).
37. SH Vosko, L Wilk, M Nusair, Accurate spin-dependent electron liquid correlation energies for local spin density calculations: a critical analysis. *Can. J. Phys.* **58**, 1200–1211 (1980).
38. C Lee, W Yang, RG Parr, Development of the Colle-Salvetti correlation-energy formula into a functional of the electron density. *Phys. Rev. B* **37**, 785–789 (1988).
39. AD Becke, Density-functional thermochemistry. III. the role of exact exchange. *J. Chem. Phys.* **98**, 5648–5652 (1993).
40. PJ Stephens, FJ Devlin, CS Ashvar, CF Chabalowski, MJ Frisch, Theoretical calculation of vibrational circular dichroism spectra. *Faraday Discuss.* **99**, 103–119 (1994).
41. TH Dunning, Gaussian basis sets for use in correlated molecular calculations. I. The atoms boron through neon and hydrogen. *The J. Chem. Phys.* **90**, 1007 (1989).
42. WR Wadt, PJ Hay, Ab initio effective core potentials for molecular calculations. potentials for main group elements na to bi. *J. Chem. Phys.* **82**, 284–298 (1985).
43. PJ Hay, WR Wadt, Ab initio effective core potentials for molecular calculations. potentials for K to au including the outermost core orbitals. *J. Chem. Phys.* **82**, 299–310 (1985).
44. PJ Hay, WR Wadt, Ab initio effective core potentials for molecular calculations. potentials for the transition metal atoms sc to hg. *J. Chem. Phys.* **82**, 270–283 (1985).
45. MJ Frisch, et al., Gaussian-09 revision e.01 (year?) Gaussian Inc. Wallingford CT 2009.
46. E Epifanovsky, et al., Software for the frontiers of quantum chemistry: An overview of developments in the Q-Chem 5 package. *J. Chem. Phys.* **155**, 084801 (2021).
47. J Shee, M Loipersberger, A Rettig, J Lee, M Head-Gordon, Regularized Second-Order Møller-Plesset theory: A more accurate alternative to conventional MP2 for noncovalent interactions and transition metal thermochemistry for the same computational cost. *J. Phys. Chem. Lett.* **12**, 12084–12097 (2021).
48. JP Perdew, Density-functional approximation for the correlation energy of the inhomogeneous electron gas. *Phys. Rev. B* **33**, 8822–8824 (1986).
49. AD Becke, Density-functional exchange-energy approximation with correct asymptotic behavior. *Phys. Rev. A* **38**, 3098–3100 (1988).
50. S Sharma, GKL Chan, Spin-adapted density matrix renormalization group algorithms for quantum chemistry. *J. Chem. Phys.* **136**, 124121 (2012).
51. Q Sun, et al., PySCF: the Python-based simulations of chemistry framework. *Wiley Interdiscip. Rev. Comput. Mol. Sci.* **8**, e1340 (2018).
52. Q Sun, et al., Recent developments in the PySCF program package. *The J. Chem. Phys.* **153**, 024109 (2020).
53. R Olivares-Amaya, et al., The ab-initio density matrix renormalization group in practice. *J. Chem. Phys.* **142**, 034102 (2015).
54. C Angeli, R Cimiraglia, S Evangelisti, T Leininger, JP Malrieu, Introduction of n-electron valence states for multireference perturbation theory. *J. Chem. Phys.* **114**, 10252 (2001).
55. S Sharma, G Knizia, S Guo, A Alavi, Combining Internally Contracted States and Matrix Product States To Perform Multireference Perturbation Theory. *J. Chem. Theory Comput.* **13**, 488–498 (2017).
56. K Raghavachari, GW Trucks, JA Pople, M Head-Gordon, A fifth-order perturbation comparison of electron correlation theories. *Chem. Phys. Lett.* **157**, 479–483 (1989).
57. JC Schöneboom, F Neese, W Thiel, Toward identification of the compound I reactive intermediate in cytochrome P450 chemistry: A QM/MM study of its EPR and mössbauer parameters. *J. Am. Chem. Soc.* **127**, 5840–5853 (2005).
58. A Altun, D Kumar, F Neese, W Thiel, Multireference ab initio quantum mechanics/molecular mechanics study on intermediates in the catalytic cycle of cytochrome P450Cam. *J. Phys. Chem. A* **112**, 12904–12910 (2008).
59. H Chen, W Lai, S Shaik, Multireference and multiconfiguration Ab initio methods in heme-related systems: What have we learned so far? *J. Phys. Chem. B* **115**, 1727–1742 (2011).
60. H Chen, J Song, W Lai, W Wu, S Shaik, Multiple low-lying states for compound i of P450cam and chloroperoxidase revealed from multireference ab initio QM/MM calculations. *J. Chem. Theory Comput.* **6**, 940–953 (2010).
61. J Lee, M Head-Gordon, Regularized Orbital-Optimized Second-Order Møller–Plesset Perturbation Theory: A Reliable Fifth-Order-Scaling Electron Correlation Model with Orbital Energy Dependent Regularizers. *J. Chem. Theory Comput.* **14**, 5203–5219 (2018).
62. CA Jiménez-Hoyos, R Rodríguez-Guzmán, GE Scuseria, Polyradical character and spin frustration in fullerene molecules: An ab initio non-collinear hartree–fock study. *The J. Phys. Chem. A* **118**, 9925–9940 (2014).
63. EG Hohenstein, RM Parrish, TJ Martínez, Tensor hypercontraction density fitting. i. quartic scaling second-and third-order møller-plesset perturbation theory. *The J. Chem. Phys.* **137**, 044103 (2012).
64. EG Hohenstein, RM Parrish, CD Sherrill, TJ Martínez, Communication: Tensor hypercontraction. iii. least-squares tensor hypercontraction for the determination of correlated wavefunctions. *The J. Chem. Phys.* **137**, 221101 (2012).
65. RM Parrish, EG Hohenstein, TJ Martínez, CD Sherrill, Tensor hypercontraction. ii. least-squares renormalization. *The J. Chem. Phys.* **137**, 224106 (2012).
66. J Lee, et al., Even more efficient quantum computations of chemistry through tensor hypercontraction. *PRX Quantum* **2**, 030305 (2021).
67. DA Matthews, Improved grid optimization and fitting in least squares tensor hypercontraction. *J. Chem. Theory Comput.* **16**, 1382–1385 (2020).
68. R Schutski, J Zhao, TM Henderson, GE Scuseria, Tensor-structured coupled cluster theory. *The J. Chem. Phys.* **147**, 184113 (2017).
69. K Pierce, V Rishi, EF Valeev, Robust approximation of tensor networks: application to grid-free tensor factorization of the coulomb interaction. *J. Chem. Theory Comput.* **17**, 2217–2230 (2021).
70. E Epifanovsky, et al., General implementation of the resolution-of-the-identity and cholesky

representations of electron repulsion integrals within coupled-cluster and equation-of-motion methods: Theory and benchmarks. *The J. Chem. Phys.* **139**, 134105 (2013).
71. BTAS: https://github.com/valeevgroup/btas (year?).

DRAFT
